# ZAR1 and ZAR2 are required for oocyte meiotic maturation by regulating the maternal transcriptome and mRNA translational activation

**DOI:** 10.1093/nar/gkz863

**Published:** 2019-10-10

**Authors:** Yan Rong, Shu-Yan Ji, Ye-Zhang Zhu, Yun-Wen Wu, Li Shen, Heng-Yu Fan

**Affiliations:** 1 MOE Key Laboratory for Biosystems Homeostasis & Protection and Innovation Center for Cell Signaling Network, Life Sciences Institute, Zhejiang University, Hangzhou 310058, China; 2 Key Laboratory of Reproductive Dysfunction Management of Zhejiang Province, Assisted Reproduction Unit, Department of Obstetrics and Gynecology, Sir Run Run Shaw Hospital, School of Medicine, Zhejiang University, Hangzhou 310016, China

## Abstract

*Zar1* was one of the earliest mammalian maternal-effect genes to be identified. Embryos derived from *Zar1*-null female mice are blocked before zygotic genome activation; however, the underlying mechanism remains unclear. By knocking out *Zar1* and its homolog *Zar2* in mice, we revealed a novel function of these genes in oocyte meiotic maturation. *Zar1/2*-deleted oocytes displayed delayed meiotic resumption and polar body-1 emission and a higher incidence of abnormal meiotic spindle formation and chromosome aneuploidy. The grown oocytes of *Zar1/2*-null mice contained decreased levels of many maternal mRNAs and displayed a reduced level of protein synthesis. Key maturation-associated changes failed to occur in the *Zar1/2*-null oocytes, including the translational activation of maternal mRNAs encoding the cell-cycle proteins cyclin B1 and WEE2, as well as maternal-to-zygotic transition (MZT) licensing factor BTG4. Consequently, maternal mRNA decay was impaired and MZT was abolished. ZAR1/2 bound mRNAs to regulate the translational activity of their 3′-UTRs and interacted with other oocyte proteins, including mRNA-stabilizing protein MSY2 and cytoplasmic lattice components. These results countered the traditional view that ZAR1 only functions after fertilization and highlight a previously unrecognized role of ZAR1/2 in regulating the maternal transcriptome and translational activation in maturing oocytes.

## INTRODUCTION

Post-transcriptional mRNA regulation is essential in the germ cells and early embryos of all animals (1–4). Oocyte meiotic maturation and early embryogenesis occur in the absence of transcription and rely on maternal mRNAs stored in oocytes (5). These maternal mRNAs generally undergo decay in embryos during the maternal-zygotic transition (MZT), in which developmental control switches from the maternal to the zygotic genome (6,7). Regulating mRNA stability thus plays a key role during these early stages of development and is tightly linked with translational regulation and mRNA localization. Recent findings in the control of mRNA stability include the essential role of deadenylation and oligo-uridylation regulation, as well as mechanisms regulating mRNA stability that involve RNA-binding proteins (8–11).

Numerous mRNAs are synthesized and stored to support oocyte maturation and early preimplantation embryogenesis and are not immediately translated (12,13). However, few maternal-effect genes that are exclusively expressed in oocytes have been identified in mammalian species (14–16). Zygote arrest-1 (*Zar1*) was identified in mice and is considered one of the earliest identified oocyte-specific maternal-effect gene that functions during MZT (17,18), other than *Mater* and *Npm2* (19,20). Female mice null for *Zar1* are infertile since embryogenesis is blocked at the 1-cell stage. This important study established *Zar1* as one of the best known mammalian maternal-effect genes, but was never independently repeated by other groups. Since then, *Zar1* has been identified in vertebrate species from zebrafish to humans; however, its precise molecular mechanism still remains unknown (21–24). Moreover, it is not yet known whether *Zar1* null oocytes are qualitatively normal before entering MZT.

More recently, a *Zar1*-like (*Zar1l*, or *Zar2*) gene was identified that is predominantly expressed in oocytes and early preimplantation embryos, shares high homology with *Zar1*, particularly the sequences encoding the C-terminal RNA-binding domain, and is conserved throughout the vertebrate lineage (25–28). Although overexpression of the ZAR2 C-terminus in mouse zygotes induced 2-cell stage arrest, the physiological function of ZAR2 has not yet been investigated *in vivo* (26).

In this study, we knocked out *Zar1* and *Zar2* in the mouse genome and investigated their potential redundant functions. While *Zar2* knockout alone caused no visible phenotypes, it strengthened *Zar1* knockout phenotypes. The double deletion of *Zar1* and *Zar2* (*Zar1/2*) impaired oocyte meiotic cell cycle progression and the translational activation of maternal mRNAs encoding important meiosis and MZT factors. ZAR1/2 also regulated the stability of the maternal transcriptome during oocyte development and MZT by binding mRNAs, interacting with other RNA-binding proteins and facilitating the accumulation of BTG4, which is a trigger of mRNA clearance during MZT. Therefore, this study revealed previously unrecognized functions of ZAR1/2 in oocyte maturation and suggested that zygotic genome activation (ZGA) failure observed in maternal *Zar1*-deleted zygotes was a consequence of defective meiotic maturation.

## MATERIALS AND METHODS

### Mice

*Zar1* and *Zar2* knockout mouse strains were made using the CRISPR/Cas9 system, as shown in Supplementary Figure S1B and C, according to experimental procedures similar to those reported previously (29). Mice were maintained under specific pathogen free (SPF) conditions in a controlled environment at 20–22°C with a 12/12 h light/dark cycle, 50–70% humidity and food and water provided *ad libitum*. All mouse strains had a C57BL6/ICR hybrid strain background. Experimental procedures and animal care conformed with the Animal Research Committee guidelines of Zhejiang University.

### Production of Cas9 and sgRNAs for *Zar1/2* knockout

The codon-optimized Cas9 expression construct, Cas9-N-NLS-flag-linker (Addgene No. 44758), was synthesized and inserted into a pST1374 vector as described previously (30). The pUC57-sgRNA expression vector used for *in vitro* sgRNA transcription was described previously (31). Oligos for generating *Zar1/2*-targeting sgRNA expression plasmids were annealed and cloned into the BsaI sites of pUC57-sgRNA.

The pST1374-Cas9-N-NLS-flag-linker vector was linearized using the AgeI enzyme and transcribed *in vitro* using a T7 Ultra Kit (Invitrogen, AM1345). SgRNA oligos were annealed into a pUC57-sgRNA expression vector with a T7 promoter, linearized by *DraI* and transcribed *in vitro* using the MEGAshortscript kit (Invitrogen, AM1354). SgRNAs were purified using a MEGAclear Kit (Invitrogen, AM1908) and recovered by alcohol precipitation. The sequences of the genotyping primers are listed in Supplementary Table S1.

### Oocyte collection and culture

Mice (21–23-day-old) were injected with 5 IU of pregnant mare serum gonadotropin (PMSG; Ningbo Sansheng Pharmaceutical, China) and humanely euthanized 44 h later. Oocytes at the GV stage were harvested in M2 medium (M7167; Sigma-Aldrich) and cultured in mini-drops of M16 medium (M7292; Sigma-Aldrich) covered with mineral oil (M5310; Sigma-Aldrich) at 37°C in a 5% CO_2_ atmosphere. In some experiments, milrinone (2 μM) was added to the culture medium to inhibit spontaneous GVBD.

### Superovulation and fertilization

Female mice (21–23-day-old) were intraperitoneally injected with 5 IU of PMSG followed by human chorionic hormone (hCG; Ningbo Sansheng Pharmaceutical, China) 44 h later. Oocytes were harvested from the oviducts 16 h after hCG injection and imaged using a Nikon SMZ1500 stereoscope. To obtain early embryos, superovulated female mice were mated with 10–12-week-old WT males. Successful mating was confirmed by the presence of vaginal plugs. Zygotes were harvested from oviducts 28 h after hCG injection.

### *In vitro* mRNA synthesis and microinjection

*In vitro* mRNA synthesis and microinjection were performed as described previously (10). Plasmids were liberalized using appropriate restriction enzymes. 5′-capped mRNAs were synthesized using Sp6 or T7 mMESSAGE mMACHINE Kits (Invitrogen, AM1340 or AM1344) and poly (A) tails were added using a Poly (A) Tailing Kit (Invitrogen, AM1350). Synthesized mRNA was recovered by lithium chloride precipitation and dissolved in nuclease-free water. All microinjections were performed using an Eppendorf transferman NK2 micromanipulator. Approximately 10 pl of synthetic RNA (∼500 μg/ml) was microinjected into the ooplasm. Oocytes were harvested in M2 medium with 2 μM milrinone to inhibit spontaneous GVBD.

### Immunofluorescence

Oocytes were fixed in 4% paraformaldehyde in phosphate-buffered saline (PBS) for 30 min and permeabilized in PBS containing 0.3% Triton X-100 for 20 min. After being blocked with 1% bovine serum albumin in PBS, the oocytes were incubated with primary antibodies for 1 h and sequentially labeled with Alexa Fluor 594- or 488-conjugated secondary antibodies (Molecular Probes) and 4′,6-diamidino-2-phenylindole (DAPI, Vector Laboratories) for 30 min. Oocytes were imaged using a Zeiss LSM710 confocal microscope. The antibodies used are listed in Supplementary Table S2.

### Detection of transcription and protein synthesis in oocytes

To detect transcriptional activity, oocytes were cultured in M16 medium containing 1 mM 5-ethynyl uridine (EU) for 1 h. EU staining was performed using a Click-iT^®^ RNA Alexa Fluor^®^ 488 Imaging Kit (Life Technologies) according to the manufacturer's instructions.

To detect protein synthesis, oocytes were cultured in M16 medium supplemented with 50 μM L-homopropargylglycine (HPG) for 1 h. HPG was detected using a Click-iT^®^ HPG Alexa Fluor^®^ Protein Synthesis Assay Kit (Life Technologies) according to the manufacturer's instructions. The mean cytoplasmic signal was measured across the middle of each oocyte and quantified using Image-J software.

### Assay with MuERVL 5′-LTR::td Tomato reporter

The plasmid containing MuERVL 5′-LTR::td Tomato reporter was previously reported and gifted by the authors (32). Zygotes were collected from oviducts of mated female mice at 24 h after hCG treatment, and microinjected with the plasmids containing MuERVL 5′-LTR::td Tomato reporter. Embryos were allowed to develop *in vitro* for another 24 h before imaging. *In vitro* transcribed and polyadenylated mRNAs encoding GFP were co-injected as a positive control reporter.

### Chromosome spreading and immunofluorescence

Zona pellucida-free oocytes were fixed in a solution containing 1% paraformaldehyde, 0.15% Triton X-100 and 3 mmol/l dithiothreitol (DTT) (Sigma-Aldrich) on glass slides for 30 min and air dried. Immunofluorescent staining was performed as described above.

### Cell culture, plasmid transfection and immunoprecipitation

HeLa cells were obtained from American tissue culture collection and cultured in Dulbecco's-modified Eagle's medium (Invitrogen) added with 10% fetal bovine serum (FBS; Hyclone) and 1% penicillin-streptomycin solution (Gibco) at 37°C with 5% CO_2_.

Mouse *Zar1/2* cDNAs were polymerase chain reaction (PCR)-amplified from a mouse oocyte cDNA pool and cloned into pCS2- or pcDNA-based eukaryote expression vectors. The cystine-to-serine mutant (4CS) of *Zar1/2* were generated by mutagenesis PCR and confirmed by sequencing. *Msy2*, *Mater* and *Padi6* clones were picked out from the ORF library of human and cloned into an N-terminal HA- or FLAG-tag vector.

Transient plasmid transfection was carried out with Lipofectamine 2000 (Invitrogen). After a 48 h transfection, cells were lysed in lysis buffer [50 mM Tris–HCl (pH 7.5), 150 mM NaCl, 10% glycerol, and 0.5% NP-40; protease inhibitors were added prior to use] for 20 min at 4°C. After centrifugation at 12 000 *g* for 10 min, the supernatant was subjected to immunoprecipitation with different affinity gels (Sigma). After incubation for 4 h at 4°C, beads were washed three times with lysis buffer. Sodium dodecyl sulphate (SDS) sample buffer was added to the beads and the eluted proteins were used for western blot analysis.

### Ribonucleoprotein immunoprecipitation (RIP) assay

The ribonucleoprotein immunoprecipitation (RIP) assay procedure was modified from a previously described method (33). Briefly, 800 fully grown oocytes for each sample or HeLa cells grown in an appropriate culture medium were collected and directly lysed in polysome lysis buffer (50 mM Tris–HCl [pH 7.4], 1% Triton X-100, 150 mM NaCl, 5 mM ethylenediaminetetraacetic acid (EDTA), protease inhibitor cocktail and RNase inhibitor). A total of 10% of the cell lysate supernatant was used as the ‘input’, while 90% was subjected to immunoprecipitation with protein-A-/-G-coated magnetic beads conjugated with IgG, ZAR1 or FLAG antibodies. After incubation at 4°C for 4 h, the beads were thoroughly washed with washing buffer (50 mM Tris–HCl [pH 7.4], 0.1% Triton X-100, 500 mM NaCl, 5 mM EDTA, protease inhibitor cocktail and RNase inhibitor). RNAs bound to the beads were extracted using an RNeasy Mini Kit (Qiagen, 74106) and reverse-transcribed using Moloney Murine Leukemia Virus (M-MLV; Invitrogen). Relative cDNA abundance was analyzed by quantitative polymerase chain reaction (qPCR).

### Real time RT-PCR

Five oocytes were collected and lysed in 2 μl lysis buffer (0.2% Triton X-100 and 2 IU/μl RNase inhibitor) followed by reverse transcription with the SuperScript III reverse transcriptase and amplification by PCR for 10 cycles. The PCR products were diluted and used for the templates of RT–PCR. Quantitative and semi-quantitative RT-PCR were performed using a Power SYBR Green PCR Master Mix (Applied Biosystems, Life technologies) with ABI 7500 Real-Time PCR system (Applied Biosystems) using primers listed in Supplementary Table S1. Relative mRNA levels were calculated by normalizing to the levels of endogenous *Actin* mRNA (internal control) using Microsoft Excel. The relative transcript levels of samples were compared with the control, and the fold changes are demonstrated. For each experiment, qPCR reactions were carried out in triplicate.

### Histological analysis

Ovaries were collected, fixed in 4% formaldehyde in PBS overnight, processed, embedded in paraffin using standard protocols, serially sectioned (5 μm), and stained with hematoxylin and eosin (H&E). Immunohistochemistry (IHC) was performed using standard protocols (34) with the antibodies listed in Supplementary Table S2.

### Western blot analysis

Oocytes were lysed in protein loading buffer and heated at 95°C for 10 min. Total oocyte proteins were separated by SDS-polyacrylamide gel electrophoresis and then electrophoretically transferred to Polyvinylidene fluoride (PVDF) membranes (Millipore). The membranes were blocked in Tris-Buffered Saline and with Tween 20 (TBST) containing 5% defatted milk for 30 min. After being probed with primary antibodies at 4°C overnight, the membranes were washed in TBST, incubated with an HRP-linked secondary antibody (Jackson ImmunoResearch Laboratories) for 1 h and washed three times with TBST. The primary antibodies are listed in Supplementary Table S2.

### RNA-seq library preparation and gene expression level analysis

Three different stage samples (10 oocytes per sample) were collected from WT and ZAR1/2-deficient mice for RNA-Seq. Growing oocytes were collected from 12-day-old female mice. GV stage oocytes were collected 44 h after PMSG injection, and MII stage oocytes were collected 16 h after hCG injection. Each sample was directly lysed with 4 μl lysis buffer (including 0.2 μl 1:1000 diluted ERCC spike-in) and immediately used for cDNA synthesis using the published Smart-seq2 method (35). Raw reads were trimmed to 50 bp and were mapped to the mouse genome (mm9) using Tophat v2.1.1 with default parameters. Only uniquely mapped reads could be subsequently assembled into transcripts guided by the reference annotation (UCSC gene models) using Cufflinks v2.2.1. The expression level of each gene was quantified with normalized FPKM (fragments per kilobase of exon per million mapped fragments) and further normalized with the ERCC spike-in. In brief, sequencing reads were mapped to ERCC reference to obtain the percentage of ERCC reads in total reads. Then gene expression levels were normalized by multiplying the raw FPKM values by a normalization factor (normalization factor = percentage of ERCC in WT Growing oocyte/percentage of ERCC in the sample). Genes with FPKM < 1 in all of the samples were excluded in subsequent analyses. For the remaining genes, the FPKM values were set to 1 when the values were <1 only in some samples. The RNA-seq data generated in this study is summarized in Supplementary Table S3. The FPKMs of the RNA-seq results are listed in Supplementary Table S4.

### Poly(A) tail assay

Total RNA was isolated from 100 oocytes at the indicated stages using an RNeasy Mini kit. R1 (5′-GCGAGCTCCGCGGCCGCGT_12_-3′) was anchored to Oligo(dT) by T4 DNA ligase. Reverse transcription was performed using SuperScript IV (Invitrogen) with Oligo(dT)-anchored R1. The products were used for PCR with gene-specific primers and dT-anchored R1 primers under the following conditions: 30 s at 94°C, 20 s at 58°C and 40 s at 72°C for 35 cycles. PCR products were analyzed on a 1.5% agarose gel and images were captured during exposure to ultraviolet light. Signals were quantified using the ‘Plot profiles’ function of ImageJ software, normalized using the maximum signal intensity in each lane, and the averaged values of three biological replicates were plotted.

### Statistical analysis

Results are given as the mean ± SEM. Most experiments included at least three independent samples and were repeated at least three times. Experimental groups were compared using two-tailed unpaired Student's *t*-tests, with statistically significant *P*-values (< 0.05, < 0.01 and < 0.001) indicated by asterisks (*, ** and ***, respectively). ‘n.s.’ indicates non-significant.

## RESULTS

### ZAR1 and ZAR2 are abundantly expressed in maturing mouse oocytes before MZT

**Figure 1.**
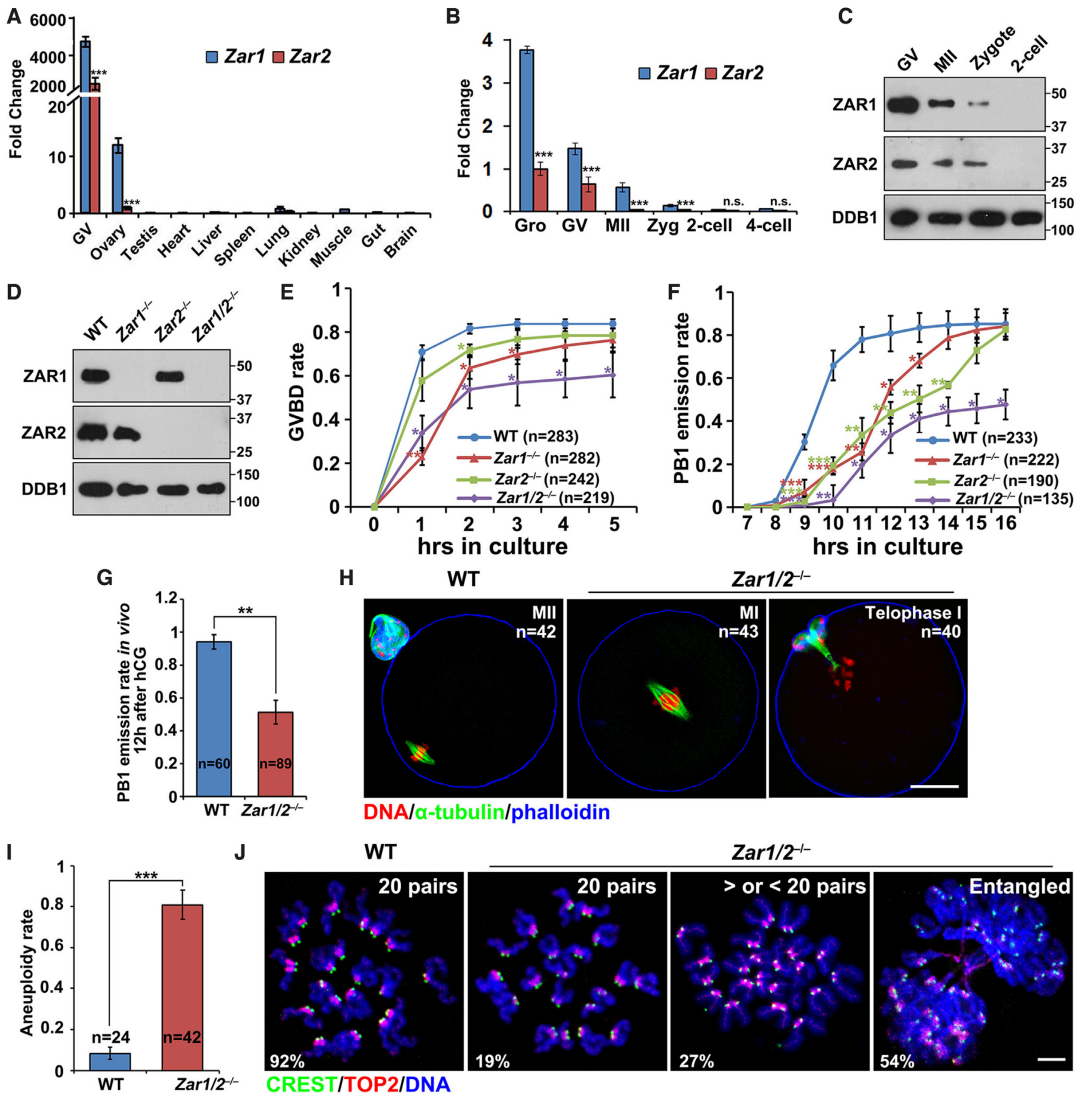
ZAR1 and ZAR2 are essential for meiotic cell cycle progression. (**A** and**B**) Quantitative RT-PCR results showing the relative expression levels of mouse *Zar1* and *Zar2* in oocytes, somatic tissues (A), and preimplantation embryos (B) (*n* = 3 biological replicates). Error bars, S.E.M. ***P < 0.001 by two-tailed Student's *t*-tests. Gro, growing oocytes collected from 12-day-old female mice. (**C**) Western blot results of ZAR1/2 levels in oocytes, zygotes and 2-cell stage embryos. Total proteins from 50 oocytes or embryos were loaded in each lane. DDB1 was blotted as a loading control. (**D**) Western blot results of ZAR1/2 levels in the GV stage oocytes of WT, *Zar1^−^^/^^−^*, *Zar2^−^^/^^−^* and *Zar1/2^−^^/^^−^* females. Total proteins from 50 oocytes were loaded in each lane. (**E** and**F**) Rates of germinal vesicle breakdown (GVBD) (E) and polar body-1 (PB1) emission (F) in oocytes cultured *in vitro*. Fully grown oocytes were collected from PMSG-primed (44 h) mice of the indicated genotypes. The numbers of analyzed oocytes are indicated (n). Error bars, S.E.M. *P < 0.05, **P < 0.01 and ***P < 0.001 by two-tailed Student's *t*-tests. (**G**) PB1 emission rates of oocytes collected from the oviducts of WT and *Zar1/2^−^^/^^−^* mice after hCG injection. The numbers of analyzed oocytes are indicated (n). Error bars, S.E.M. **P < 0.01 by two-tailed Student's *t*-tests. (**H**) Confocal microscopy results showing spindle assembly and PB1 emission of oocytes collected from the oviducts of WT and *Zar1/2^−^^/^^−^* mice. Scale bar, 20 μm. The numbers of analyzed oocytes are indicated (n). (**I**) Aneuploidy rates of oocytes collected from the oviducts of WT and *Zar1/2^−^^/^^−^* mice 12 h after hCG injection. The numbers of analyzed oocytes are indicated (n). Error bars, S.E.M. ***P < 0.001 by two-tailed Student's *t*-tests. (**J**) Representative chromosome spread images of oocytes collected from the oviducts of WT and *Zar1/2^−^^/^^−^* mice. Immunofluorescent staining of topoisomerase II (TOP2) and the centromere antigen CREST indicated the chromosome arms and centromeres, respectively. The numbers of paired sister chromatids are indicated in the top right-hand corner. Scale bar, 5 μm.

We compared *Zar1*/Z*ar2* mRNA levels in oocytes and early embryos by quantitative RT–PCR. *Zar1*/*Zar2* mRNAs were highly expressed in mouse oocytes compared to somatic tissues (Figure 1A). *Zar1/2* mRNA levels were highest in growing oocytes, lower in fully grown oocytes at the germinal vesicle (GV) stage, and quickly disappeared after meiotic maturation and fertilization (Figure 1B). *Zar1* was 2- to 4-fold more abundant than *Zar2* (Figure 1A and B). We generated rabbit polyclonal antibodies against mouse ZAR1 and ZAR2, with IHC revealing that ZAR1 protein levels were very low in the oocytes of primordial follicles (Supplementary Figure S1A, indicated by the arrow) but quickly increased after primordial follicle awakening (Supplementary Figure S1A). The specificity of the ZAR1 antibody in IHC was confirmed on ovarian sections from *Zar1* knockout mice (Supplementary Figure S1A). The ZAR2 antibody did not give specific IHC signals (data not shown); however, western blotting showed that ZAR1/2 protein levels were high in GV oocytes, decreased during meiotic maturation and fertilization, and undetectable in 2-cell embryos (Figure 1C). Their accumulation during oocyte growth and degradation after meiotic resumption suggest that ZAR1/2 play a role during oocyte maturation before fertilization.

### *Zar1* and *Zar2* are essential for oocyte meiotic maturation

Prior to this study the *in vivo* functions of *Zar2* had not been reported; therefore, we generated a *Zar2* knockout mouse strain using the CRISPR/Cas9 system. The *Zar2* knockout strain contained a 152 bp deletion in exon 1 after the start codon that caused a reading frame shift thereafter (Supplementary Figure S1B). ZAR2 deletion was confirmed in oocytes by western blotting (Figure 1D). Although *Zar2* is specifically expressed in female germ cells, *Zar2^–/–^* females were fertile (Supplementary Figure S2A). We isolated fully grown GV oocytes from pregnant mare serum gonadotropin (PMSG)-primed *Zar2^–/–^* females and cultured the oocytes *in vitro*. The germinal vesicle breakdown (GVBD) process of *Zar2* null oocytes was slightly slower than normal (Figure 1E), but their polar body-1 (PB1) emission occurred more slowly than in wild-type (WT) oocytes (Figure 1F). These defects did not affect fertility since the GVBD and PB1 emission rates of *Zar2^–/–^* oocytes caught up with the WT oocytes, albeit at a later time. This phenotype suggests that ZAR2 plays a role in oocyte meiotic maturation, but its loss can be compensated by other oocyte factors, most likely ZAR1.

To determine whether ZAR1 plays a redundant role with ZAR2, we generated a novel *Zar1* knockout stain using the CRISPR/Cas9 system. The *Zar1* null allele contained a 12 bp deletion followed by a stop codon shortly after the translational start site (Supplementary Figure S1C). ZAR1 protein deletion in the oocytes of these mice was confirmed by both western blotting and IHC (Figure 1D and Supplementary Figure S1A). *Zar1^–/–^* females were sterile (Supplementary Figure S2A), as reported (17); however, we observed additional phenotypes in these mice that had not been reported before: *Zar1* null oocytes displayed delayed GVBD and PB1 emission *in vitro* (Figure 1E and F). When both *Zar1* and *Zar2* were deleted, GVBD and PB1 emission rates in the cultured oocytes decreased significantly, even with a prolonged culture time (Figure 1E and F; Supplementary Figure S2B).

Next, we investigated the *in vivo* oocyte maturation process in *Zar1/2^–/–^* mice. In females treated with PMSG to stimulate follicle growth (Supplementary Figure S2C) and non-treated adults (Supplementary Figure S2D), ovarian histology was normal and contained developing follicles of all stages. The ratio of GV oocytes containing surrounded nucleolus (SN) versus non-surrounded nucleolus (NSN) in *Zar1/2^–/–^* mice (3-week-old) was comparable with WT mice of the same age (Supplementary Figure S2E). The *Zar1/2^–/–^* mice ovulated normal numbers of oocytes following superovulation treatment (Supplementary Figure S2F). We collected oocytes from the oviducts and analyzed their potential defects. Similar to the *in vitro* maturation results, only 51% of the *Zar1/2^–/–^* oocytes exhibited PB1 emission, as observed using a stereoscope (Figure 1G). Immunofluorescence and confocal microscopy showed that the *Zar1/2^–/–^* oocytes failed to release PB1 and arrested at metaphase I (Figure 1H). Furthermore, the *Zar1/2^–/–^* oocytes that seemed to have released PB1 were actually at meiosis I telophase: cytokinesis had not finished, PB1 was still connected with the oocyte via a cytoplasmic bridge, and the MII spindle had not assembled in all oocytes (Figure 1H). We collected these oocytes, made chromosome spreads and labeled the chromosomes by immunostaining with CREST (localized at the centromere) and topoisomerase II (TOP2, localized on chromosome arms, particularly the region adjacent to the centromere) (36). When 92% of the WT oocytes contained 20 pairs of sister chromatids, the telophase I-arrested *Zar1/2^–/–^* oocytes showed high rates of aneuploidy, either having more or less than 20 chromatid pairs (27%) or containing entangled and unseparated chromosome masses (54%; Figure 1I and J).

### *Zar1/2^–/–^* oocyte-derived embryos arrest at the 1- or 2-cell stage due to meiosis defects

**Figure 2.**
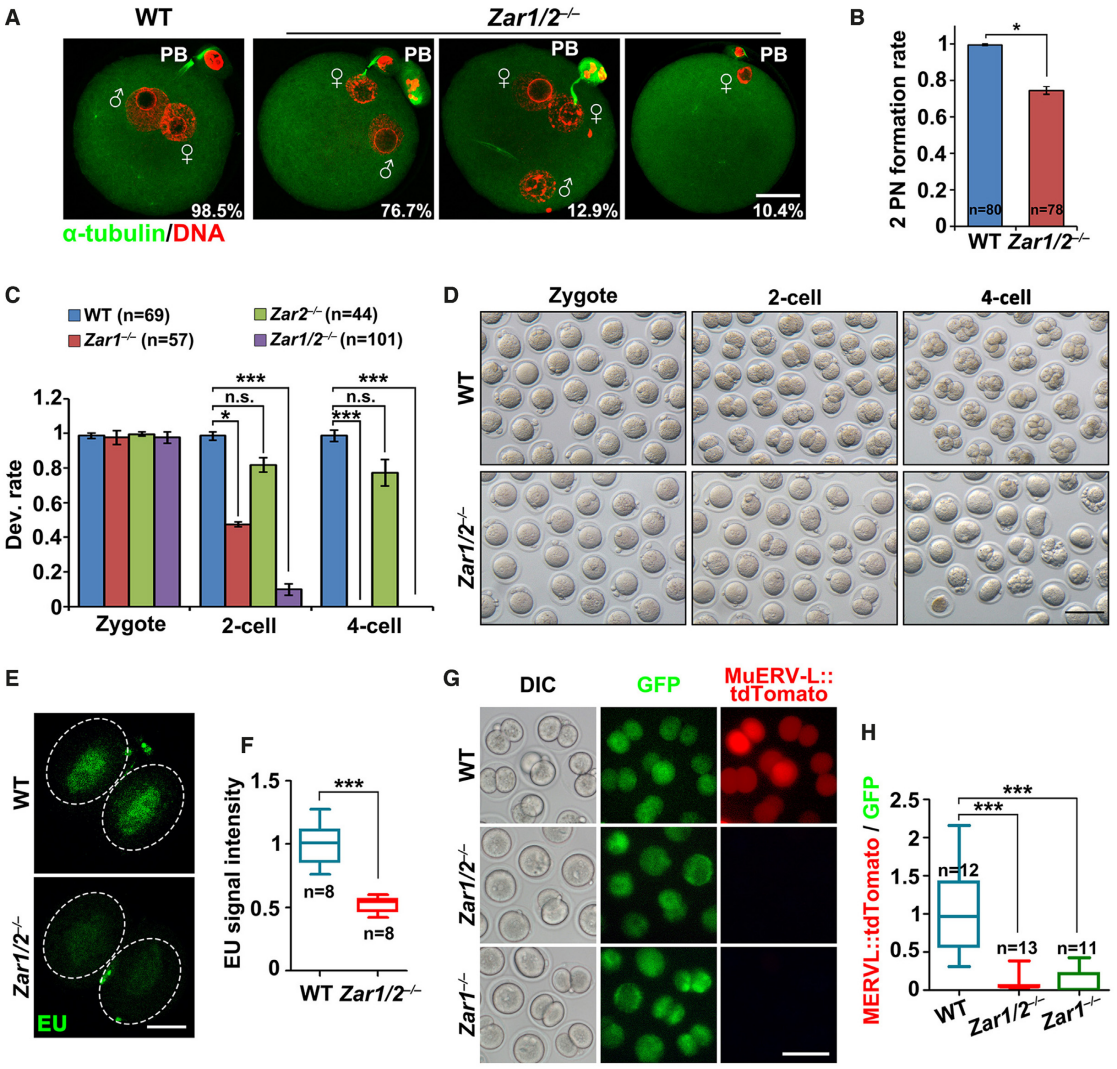
*Zar1/2* deletion in oocytes causes zygote arrest. (**A**) Immunofluorescent staining for α-tubulin (green) and DNA (red) in zygotes from WT and *Zar1/2^−^^/^^−^* females 28 h after hCG injection. Scale bar, 20 μm. (**B**) Percentage of zygotes with two pronuclei (PNs) from WT and *Zar1/2^−^^/^^−^* females 28 h after hCG injection. The number of analyzed oocytes is indicated (n). Error bars, S.E.M. *P < 0.05 by two-tailed Student's *t*-tests. (**C**) Quantification of preimplantation embryos derived from WT and *Zar1/2^−^^/^^−^* females when WT embryos reached the corresponding stages. The number of analyzed embryos is indicated (n). *P < 0.05 and ***P < 0.001 by two-tailed Student's *t*-tests. n.s.: non-significant. (**D**) Representative images of preimplantation embryos derived from WT and *Zar1/2^−^^/^^−^* females when WT embryos reached the corresponding stages. Scale bar, 100 μm. (**E**) EU fluorescent staining for the transcriptional activity of mRNA in 2-cell stage embryos from WT and *Zar1/2^−^^/^^−^* females 44 h after hCG injection. Embryos were incubated in M16 medium with 1 mM EU for 1 h prior to staining. Scale bars, 20 μm. (**F**) Quantification of EU signal intensity in (E). The number of analyzed embryos is indicated (n). Error bars, SEM. ***P < 0.001 by two-tailed Student's *t*-tests. (**G**) Zygotes were injected with the MuERV-L::tdTomato reporter plasmid and polyadenylated *Gfp* mRNA (as a positive control of microinjection), then allowed to develop *in vitro* for 24 h before imaging. DIC, differential interference contrast. Scale bar, 100 μm. (**H**) Relative intensity of MuERV-L::tdTomato relative to GFP signal in the same oocyte (G). The numbers of analyzed embryos are indicated (n). Error bars, S.E.M. ***P < 0.001 by two-tailed Student's *t*-tests.

We mated superovulated *Zar1/2^–/–^* female mice with WT males. Despite PB1 emission and MII spindle assembly defects, *Zar1/2^–/–^* oocytes were fertilized, as characterized by pronucleus formation (Figure 2A). All control zygotes contained two pronuclei 28 h post-hCG injection; however, zygotes derived from *Zar1/2*-null oocytes contained one or more nuclei (Figure 2A and B). While the embryos of WT and *Zar2^–/–^* females developed normally, *Zar1^–/–^* female-derived embryos (*Zar1^♀−^^/^^♂^^+^*) arrested at the 1- or 2-cell stage (50% each). Over 90% of the *Zar1/2^♀−^^/^^♂^^+^* zygotes arrested at the 1-cell stage and none developed beyond the 2-cell stage (Figure 2C and D), with EU staining revealing that overall transcriptional activation in the 2-cell embryos was impaired by maternal *Zar1/2* knockout (Figure 2E and F).

In addition, a feature of 2-cell embryos undergoing ZGA is the expression of MuERV-L family of retrovirus and the activation of their corresponding long terminal repeat (LTR) promoters (32). We microinjected zygotes (WT, *Zar1^♀−^^/^^♂^^+^* and *Zar1/2^♀−^^/^^♂^^+^*) with the MuERV-L 5′-LTR::tdTomato reporter plasmid, and monitored the expression of tdTomato during development. TdTomato expression was only detected in WT 2-cell embryos, but not in *Zar1^♀−^^/^^♂^^+^* and *Zar1/2^♀−^^/^^♂^^+^*embryos (Figure 2G and H). In contrast, *Gfp* mRNAs co-injected with the MuERV-L 5′-LTR reporter were equally expressed in all embryos (Figure 2G). These results further indicate that ZGA failed to occur in maternal *Zar1/2* deleted embryos.

### ZAR1/2 deletion impaired maternal mRNA accumulation during oocyte development

Since ZAR1/2 are predicted RNA-binding proteins (27,28), we investigated the effects of maternal *Zar1/2* deletion on mRNA metabolism during oocyte maturation. We subjected growing oocytes, fully grown GV oocytes and ovulated oocytes from WT and *Zar1/2^–/–^* mice to transcriptome RNA-seq analyses by smart-seq 2. Gene expression levels were assessed in terms of fragments per kilobase of transcript per million mapped reads (FPKM). All experimental and control groups were analyzed in duplicate and all replicates showed high correlation (Supplementary Figure S3; *R*_min_ = 0.88; *R*_average_ = 0.93; Supplementary Table S5).

**Figure 3.**
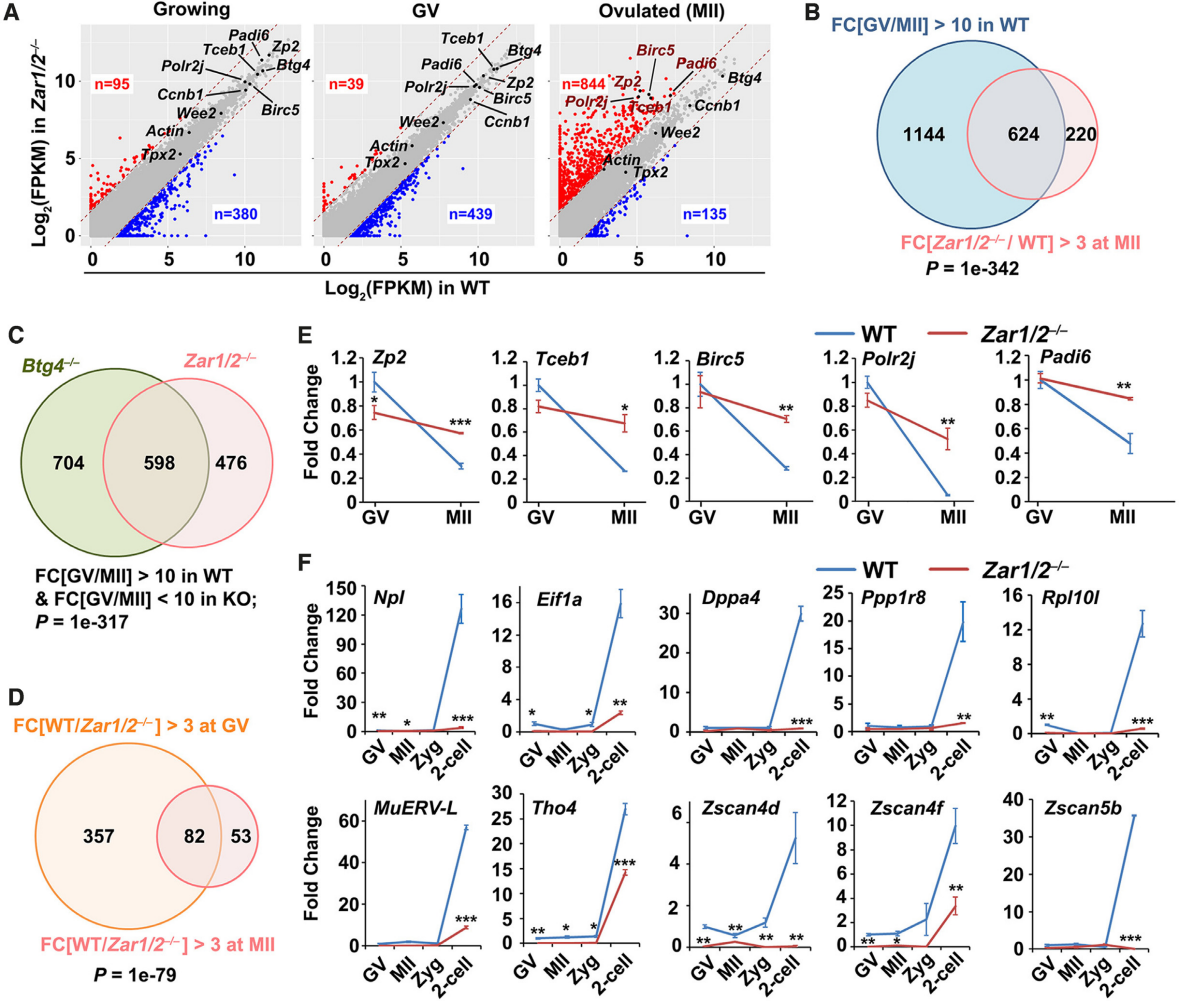
RNA sequencing analyses of oocytes derived from WT and *Zar1/2^−^^/^^−^* female mice. (**A**) Scatter plot comparing the transcripts of WT and *Zar1/2^−^^/^^−^* oocytes (growing, fully grown GV and MII stages). Transcripts that increased or decreased by more than 3-fold in *Zar1/2^−^^/^^−^* oocytes are highlighted in red or blue, respectively. (**B**) Venn diagrams showing the overlap in transcripts that were significantly degraded during GV–MII transition (fold change (GV/MII) > 10) of WT oocytes and genes upregulated in this process after *Zar1/2* knockout (fold change (*Zar1/2^−^^/^^−^*/WT in MII) > 3). (**C**) Venn diagrams showing the overlap in transcripts that were stabilized during GV-to-MII transition in *Zar1/2^−^^/^^−^* and *Btg4^−^^/^^−^* oocytes (fold change (GV/MII) > 10 in WT, but < 10 in KO). (**D**) Venn diagrams showing the overlap in transcripts that were downregulated (fold change (WT/ *Zar1/2^−^^/^^−^*) > 3) at the GV and MII stages after *Zar1/2* knockout. (**E**) Quantitative RT–PCR results for relative levels of the indicated maternal transcripts in oocytes at GV and MII stages from WT and *Zar1/2^−^^/^^−^* females. *n* = 3 biological replicates. Error bars, SEM. *P < 0.05, **P < 0.01 and ***P < 0.001 by two-tailed Student's *t*-tests. (**F**) Quantitative RT–PCR results for the relative levels of indicated transcripts in the oocytes and embryos of WT and *Zar1/2^−^^/^^−^* females. *n* = 3 biological replicates. Error bars, SEM. *P < 0.05, **P < 0.01 and ***P < 0.001 by two-tailed Student's *t*-tests.

Although the growing *Zar1/2^–/–^* oocytes were morphologically normal, 95 and 380 transcripts were upregulated [fold change (WT/*Zar1/2^–/–^*) < 1/3] and downregulated [fold change (WT/*Zar1/2^–/–^*) > 3], respectively (Figure 3A, left panel). This effect became more apparent in fully grown *Zar1/2^–/–^* oocytes, in which more genes were downregulated (439) than upregulated (37; Figure 3A, middle panel). The levels of 844 transcripts were higher in ovulated *Zar1/2^–/–^* oocytes than in WT oocytes (Figure 3A, right panel). The significantly up/downregulated genes after *Zar1/2* knockout at each developmental stage were presented in Supplementary Table S6. The majority of these transcripts should normally be degraded during oocyte maturation; however, their clearance was impaired in *Zar1/2^–/–^* oocytes (Figure 3B). Figure 3C showed the significant overlap between maternal transcripts whose degradation during meiotic maturation is *Zar1/2*-dependent and *Btg4*-dependent, the latter were identified in a previous study (10). Conversely, 135 transcripts exhibited lower copy numbers in ovulated *Zar1/2^–/–^* oocytes than in control oocytes (Figure 3A, right panel), with the majority of these transcripts also exhibiting decreased expression levels at the onset of meiotic maturation (Figure 3D). These results indicated that ZAR1 and ZAR2 regulate the maternal transcriptome during oocyte development, before MZT.

Using RT-qPCR, we confirmed the RNA-seq results and demonstrated that the selected maternal transcripts were degraded during WT oocyte maturation but were stabilized by *Zar1/2* knockout (Figure 3E). The mRNA levels in the different samples were normalized to the housekeeping gene *Actin*, whose expression level remains unchanged during oocyte development and is unaffected by *Zar1/2* knockout (Figure 3A). Known early zygotic genes were expressed at very low levels in WT oocytes and zygotes but accumulated at the 2-cell stage (Figure 3F). RT–qPCR indicated that the transcriptional activation of these genes was blocked in *Zar1/2^♀−^^/^^♂^^+^* embryos (Figure 3F). These genes included the endogenous retrovirus MuERV-L and *Zscan4* family members which maintain totipotency in 2-cell embryos and 2-cell-like stem cells (32,38), as well as other early zygotic genes (*Npl*, *Eif1a*, *Dppa4*, *Ppp1r8* and *Rpl10l*) with undetermined functions in MZT. These results further indicate that global ZGA was blocked by maternal *Zar1/2*-deletion.

Since the *Zar1/2^–/–^* oocytes had meiosis defects and arrested in metaphase I or telophase I, we could not conclude whether the mRNA degradation defect was primarily due to a lack of ZAR1/2 or a secondary consequence of arresting oocytes in meiosis I. To address this question, we artificially disrupted spindle assembly and arrested maturing oocytes in meiosis I by treatment with nocodazole (100 ng/ml; Supplementary Figure S4A and B), a widely used microtubule disruptor. We then detected selective mRNA degradation in these oocytes by RT-qPCR, showing that mRNA degradation was not affected by nocodazole treatment (Supplementary Figure S4C). These results strongly suggest that the delayed mRNA decay observed in *Zar1/2* null oocytes was due to a lack of ZAR1/2 rather than a secondary consequence of arresting oocytes in meiosis I.

### ZAR1/2-deletion impaired maternal mRNA translation during oocyte meiotic maturation

The decrease in maternal transcripts in ZAR1 and ZAR2 deleted oocytes may be due to decreased transcription or increased mRNA degradation, or both. To verify these possibilities, we assessed the global transcription activities of growing oocytes (isolated from 12-day-old mice) using a 5′-ethynyl uridine (EU) incorporation assay. The signals showed no significant differences between growing WT and *Zar1/2*-null oocytes (Supplementary Figure S5A and B). Similar results were obtained from NSN oocytes isolated from 23-day-old WT and *Zar1/2*-null mice (Supplementary Figure S5C and D). In contrast, EU signals were not detected in fully grown WT and *Zar1/2*-null oocytes with a SN chromosome configuration (Supplementary Figure S5C and D). These results indicate that: (i) *Zar1/2* knockout does not affect transcriptional silencing in fully grown oocytes; and (ii) the EU signal detected in growing oocytes is specific.

**Figure 4.**
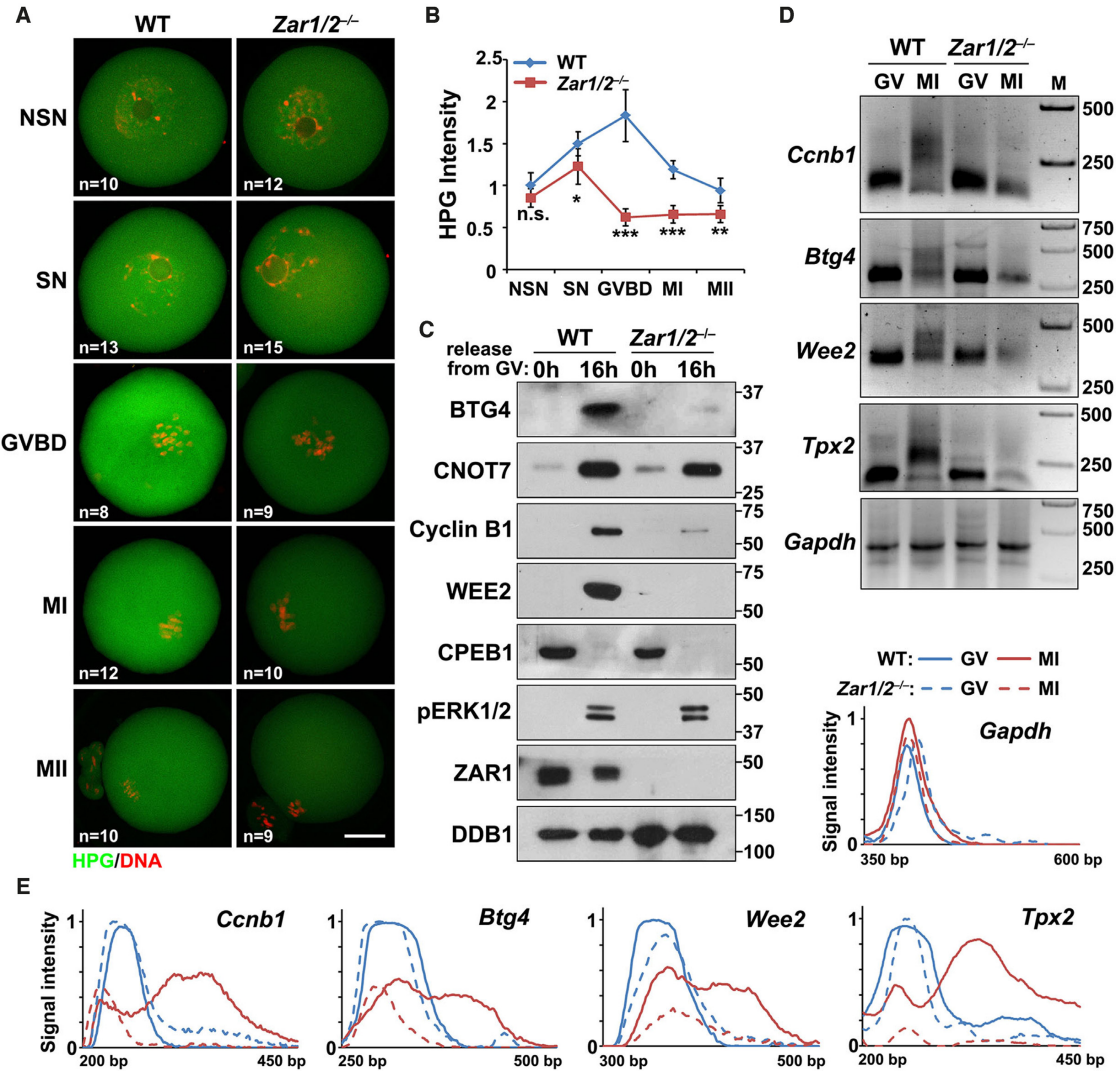
Low protein translation impairs meiotic maturation in *Zar1/2*-deleted oocytes. (**A**) HPG fluorescent staining shows protein synthesis activity in oocytes at the indicated stages. Oocytes were incubated in M16 medium with 50 μM HPG for 1 h prior to staining. The number of analyzed oocytes at each stage is indicated (n). Scale bars, 20 μm. (**B**) Quantification of HPG signal intensity in (A). Error bars, SEM. *P < 0.05, **P < 0.01 and ***P < 0.001 by two-tailed Student's *t*-tests. n.s.: non-significant. (**C**) Western blot results showing the expression levels of the indicated proteins in the GV and MII oocytes of WT and *Zar1/2^−^^/^^−^* females. Total proteins from 100 oocytes were loaded in each lane. (**D**) Changes in poly(A)-tail length of the indicated transcripts in GV and MII oocytes collected from WT and *Zar1/2^−^^/^^−^* females. The poly(A) tails of *Gapdh* were used as an internal control. (**E**) Quantification of the PAT assay results in (**D**). Plots show the averaged relative signal intensity (*y*-axis) and length of the PCR products based on mobility (*x*-axis).

Considering that *Zar1/2* deletion would probably affect global protein synthesis, we incubated *Zar1/2^–/–^* oocytes with L-HPG, a methionine analogue. In the control groups, fully grown SN oocytes had stronger HPG signals than the growing NSN oocytes, indicating that total protein synthesis increased during oocyte development (Figure 4A and B). Protein translation activity further increased after meiotic resumption and then decreased during MI and MII. These changes reflected the translational activation of dormant maternal transcripts at the onset of meiotic resumption followed by the prompt degradation of these transcripts. In comparison, the *Zar1/2^–/–^* oocytes had lower HPG signals than the control oocytes, particularly during pro-MI (Figure 4A and B).

Some proteins that normally accumulate after meiotic resumption, including BTG4 (10,39), cyclin B1 (37) and WEE2 (40), were not expressed in ovulated *Zar1/2^–/–^* oocytes (Figure 4C). Nevertheless, biochemical events upstream of meiotic resumption-coupled mRNA translational activation, namely ERK1/2 phosphorylation and CPEB1 degradation (13), occurred normally in *Zar1/2^–/–^* oocytes. CNOT7 is the catalytic subunit of CCR4-NOT deadenylase, which interacts with BTG4 to mediate maternal mRNA decay (10,41); its mRNA also undergoes translational activation during meiotic maturation. Unlike BTG4, CNOT7 accumulation was not impaired in *Zar1/2^–/–^* oocytes (Figure 4C). At the GV stage, the maternal mRNAs encoding BTG4, cyclin B1 and WEE2 were at lower levels in *Zar1/2^–/–^* oocytes than those in WT oocytes, but in MII oocytes from WT and *Zar1/2^–/–^* mice, these mRNAs were at comparable levels (Figure 3A, gene names are indicated in the charts; Supplementary Figure S6A). Therefore, we hypothesized that the translational activation of certain maternal mRNAs was impaired in *Zar1/2* null oocytes.

For dormant maternal mRNAs, poly(A) tail elongation is the key step in translational activation (42,43). Therefore we assessed the poly(A) tail length of mRNAs encoding cyclin B1, BTG4 and WEE2 using a poly(A) tail (PAT) assay (Figure 4D and E). In GV stage WT oocytes, these mRNAs contained short tails but underwent remarkable polyadenylation at the MI stage. In contrast, the meiotic resumption-coupled tail elongation of these transcripts failed in *Zar1/2^–/–^* oocytes. The level and poly(A) length of the *Gapdh* transcript, used as an internal control, remained unchanged in all samples (Figure 4D and E).

### ZAR1/2-deletion impaired the 3′-UTR activation of maternal mRNAs encoding proteins essential for meiotic maturation and MZT

**Figure 5.**
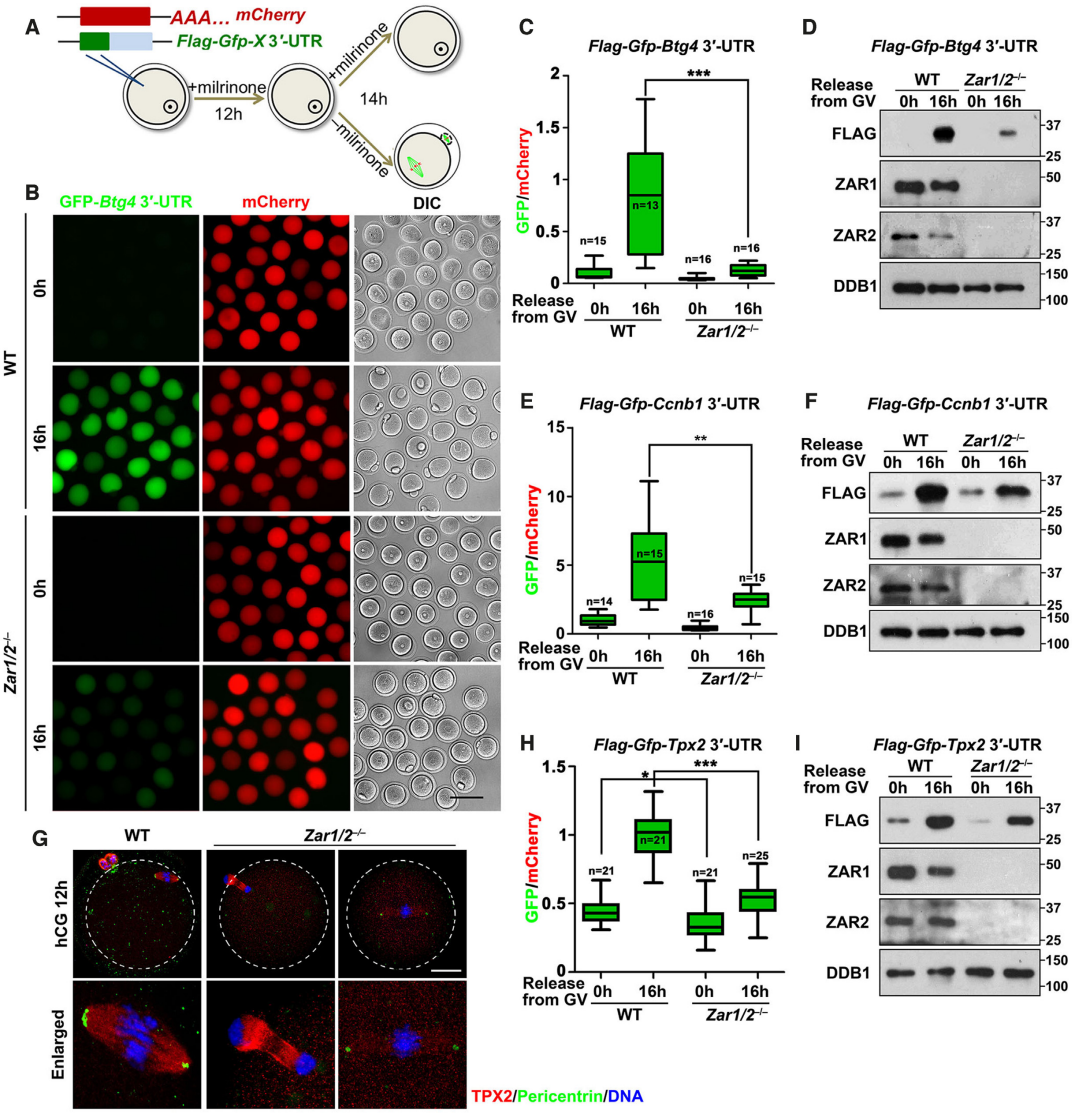
ZAR1/2 regulate the translational activation of maternal mRNAs. (**A**) Diagram of the 3′-UTR reporter assay. (**B**) Fluorescence microscopy showing the expression of FLAG-GFP fused with *Btg4* 3′-UTR in WT and *Zar1/2^−^^/^^−^* oocytes. Scale bar, 100 μm. (**C** and **D**) Relative GFP intensity (C) and western blot analysis (D) showing the expression of FLAG-GFP fused with *Btg4* 3′-UTR in WT and *Zar1/2^−^^/^^−^* oocytes. (**E** and **F**): Relative GFP intensity (E) and western blot analysis (F) showing the expression of FLAG-GFP fused with *Ccnb1* 3′-UTR in WT and *Zar1/2^−^^/^^−^* oocytes. (**G**) TPX2 immunofluorescence of oocytes from WT and *Zar1/2^−^^/^^−^* females 12 h after hCG injection. MTOCs and DNA were labeled with pericentrin and DAPI, respectively. Scale bar, 20 μm. (**H** and**I**) Relative GFP intensity (H) and western blot (I) analysis showing the expression of FLAG-GFP fused with *Tpx2* 3′-UTR in WT and *Zar1/2^−^^/^^−^* oocytes. For all panels, the number of analyzed oocytes is indicated (n). Error bars, S.E.M. *P < 0.05, **P < 0.01 and ***P < 0.001 by two-tailed Student's *t*-tests. Total proteins from 100 oocytes were loaded in each lane.

To determine whether ZAR1/2 played a role in the translational activation of *Btg4* and *Ccnb1* mRNAs, we constructed Flag-GFP reporters fused with the 3′-untranslated region (3′-UTR) of mouse *Btg4* (10,12–13). These reporter mRNAs were transcribed *in vitro* and microinjected into GV stage-arrested WT and *Zar1/2^–/–^* oocytes, with an *in vitro* polyadenylated mCherry transcript coinjected as a positive control (Figure 5A). After 16 h in the WT groups, we detected weak GFP signals in GV-arrested oocytes and strong GFP signals in MII oocytes, whereas the mCherry signals were equal in the GV- and MII-arrested oocytes (Figure 5B and C). The translation of Flag-GFP driven by *Btg4* 3′-UTR was also detected in the mature WT oocytes by western blot, but was remarkably reduced in *Zar1/2^–/–^* oocytes (Figure 5D). Similar results were obtained using a *Ccnb1* 3′-UTR (Figure 5E and F; Supplementary Figure S6B). These results indicate that the decreased BTG4 and cyclin B1 levels in maturing *Zar1/2^–/–^* oocytes were primarily due to insufficient translational activation of their 3′-UTRs.

TPX2 (targeting protein for the *Xenopus* kinesin xKLP2) is required for spindle assembly during oocyte maturation and *Tpx2* mRNAs undergo translational activation after meiotic resumption (44,45). The *Tpx2* mRNA levels in *Zar1/2* null oocytes were comparable to those in WT oocytes throughout development (Figure 3A; Supplementary Figure S6A). TPX2 localized to the meiotic spindles of WT oocytes and pericentrin, a marker of the microtubule organizing center (MTOC), was visualized as dots concentrated at the spindle poles (Figure 5G). The TPX2 signal was low and diffuse on the spindles of *Zar1/2* null oocytes, whereas the pericentrin dots were smaller and their fluorescent signals were weaker than in WT oocytes (Figure 5G). Although the TPX2 antibody sensitivity was not high enough to detect endogenous TPX2 by western blotting (data not shown), PAT (Figure 4D and E) and 3′-UTR reporter (Figure 5H and I; Supplementary Figure S6C) assays indicated that *Tpx2* mRNA polyadenylation and translation failed during oocyte maturation. Impaired TPX2 accumulation might cause spindle assembly and PB1 emission failures in *Zar1/2^–/–^* oocytes.

To rule out the possibility that impaired spindle assembly and PB1 emission in *Zar1/2^–/–^* oocytes might nonspecifically affect the translational activation of maternal mRNAs, we evaluated the protein translational activity of spindle-disrupted (nocodazole treatment) WT oocytes. HPG staining indicated that global translation activity was similar in oocytes cultured for 16 h in medium with (arrested at MI) and without (released PB1 and developed to MII) nocodazole (Supplementary Figure S4D and E). The *Btg4* 3′-UTR reporter experiment also showed that *Btg4* 3′-UTR translational activity was not affected by spindle disruption (Supplementary Figure S4F–H).

### ZAR1/2 interact with mRNAs via their C-terminal zinc-finger domains

**Figure 6.**
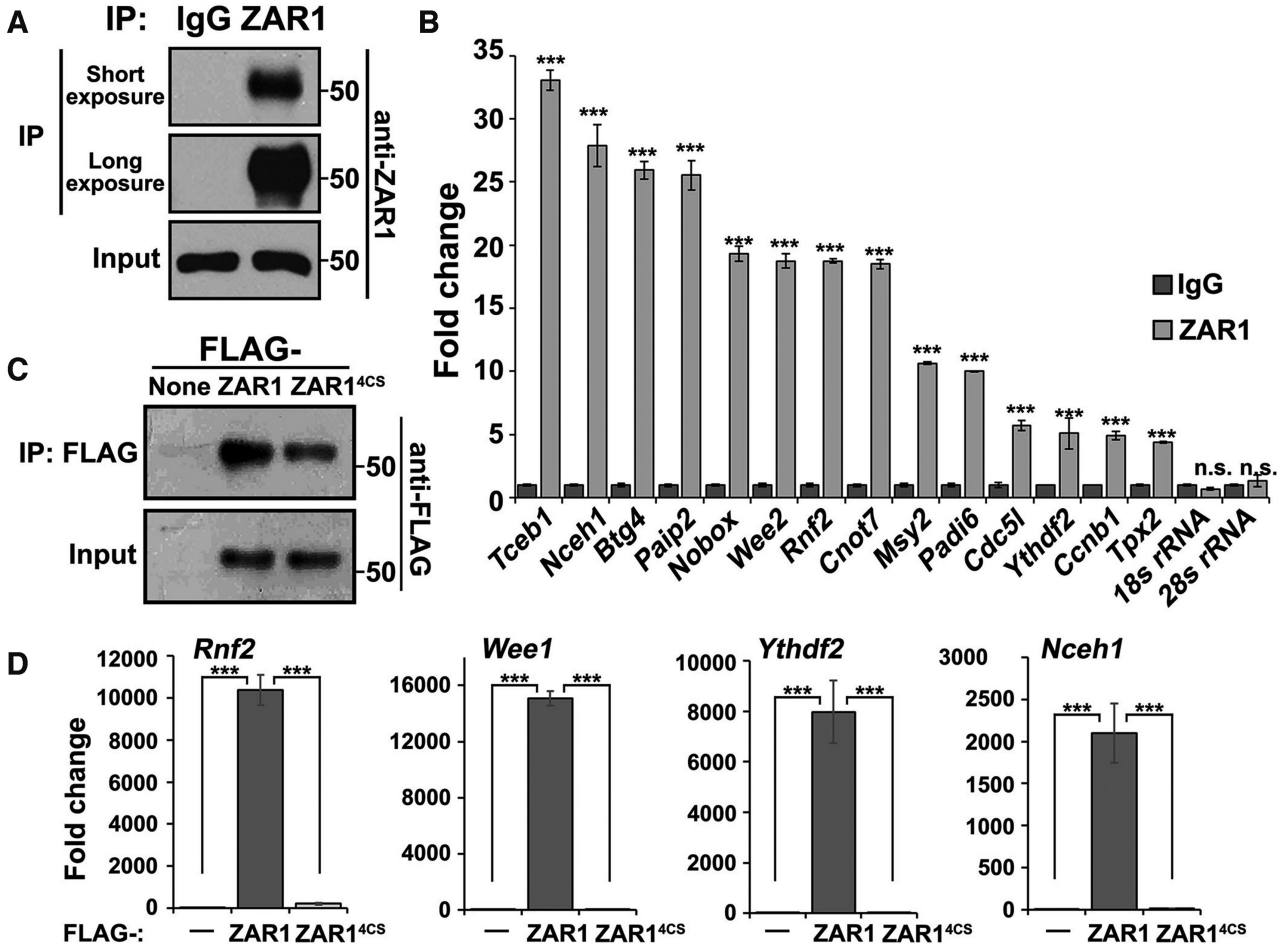
ZAR1/2 interact with mRNAs via their C-terminal zinc-finger domains. (**A**) Western blot results showing that the endogenous ZAR1 protein was specifically pulled down from WT oocyte lysates by an anti-ZAR1 antibody. (**B**) RIP results using an anti-ZAR1 antibody showing the interactions between ZAR1 and maternal transcripts in GV oocytes. Levels of mRNAs coprecipitated with ZAR1 were detected by RT-qPCR. *n* = 3 biological replicates. Error bars, S.E.M. ***P < 0.001 by two-tailed Student's *t*-tests. n.s.: non-significant. (**C**) Western blot results showing that Flag-tagged ZAR1 and ZAR1^4CS^ were specifically pulled down from HeLa cell lysates using an anti-FLAG antibody. (**D**) RIP assay results using an anti-FLAG antibody showing the interactions between ZAR1 (FLAG-ZAR1 and FLAG-ZAR1^4CS^) and the indicated transcripts in HeLa cells. The levels of mRNAs coprecipitated with ZAR1 were detected by RT-qPCR. n = 3 biological replicates. Error bars, S.E.M. ***P < 0.001 by two-tailed Student's *t*-tests.

Next, we investigated whether ZAR1/2 physically interact with mRNAs to regulate their translation using RNA immunoprecipitation (RIP) assays. Endogenous ZAR1 proteins were specifically pulled down from WT oocyte lysates using an anti-ZAR1 antibody (Figure 6A). RT-qPCR demonstrated that maternal transcripts encoding proteins essential for oocyte function were enriched in the ZAR1 precipitates (Figure 6B). We also overexpressed WT and RNA-binding domain-mutated ZAR1 (ZAR1^4CS^) in HeLa cells, which contain no endogenous ZAR1 proteins. Immunoprecipitation and RT-qPCR showed that select transcripts (which were also found to be expressed in oocytes by RNA-seq) physically bind with ZAR1, but not ZAR1^4CS^ (Figure 6C and D). Similar results were obtained by RIP assays using ZAR2 (Supplementary Figure S7A–C). Therefore, ZAR1/2 physically interact with mRNAs and their RNA binding ability relies on their C-terminal zinc finger domains.

### ZAR1/2 interact with MSY2 and are localized to cytoplasmic lattices

**Figure 7.**
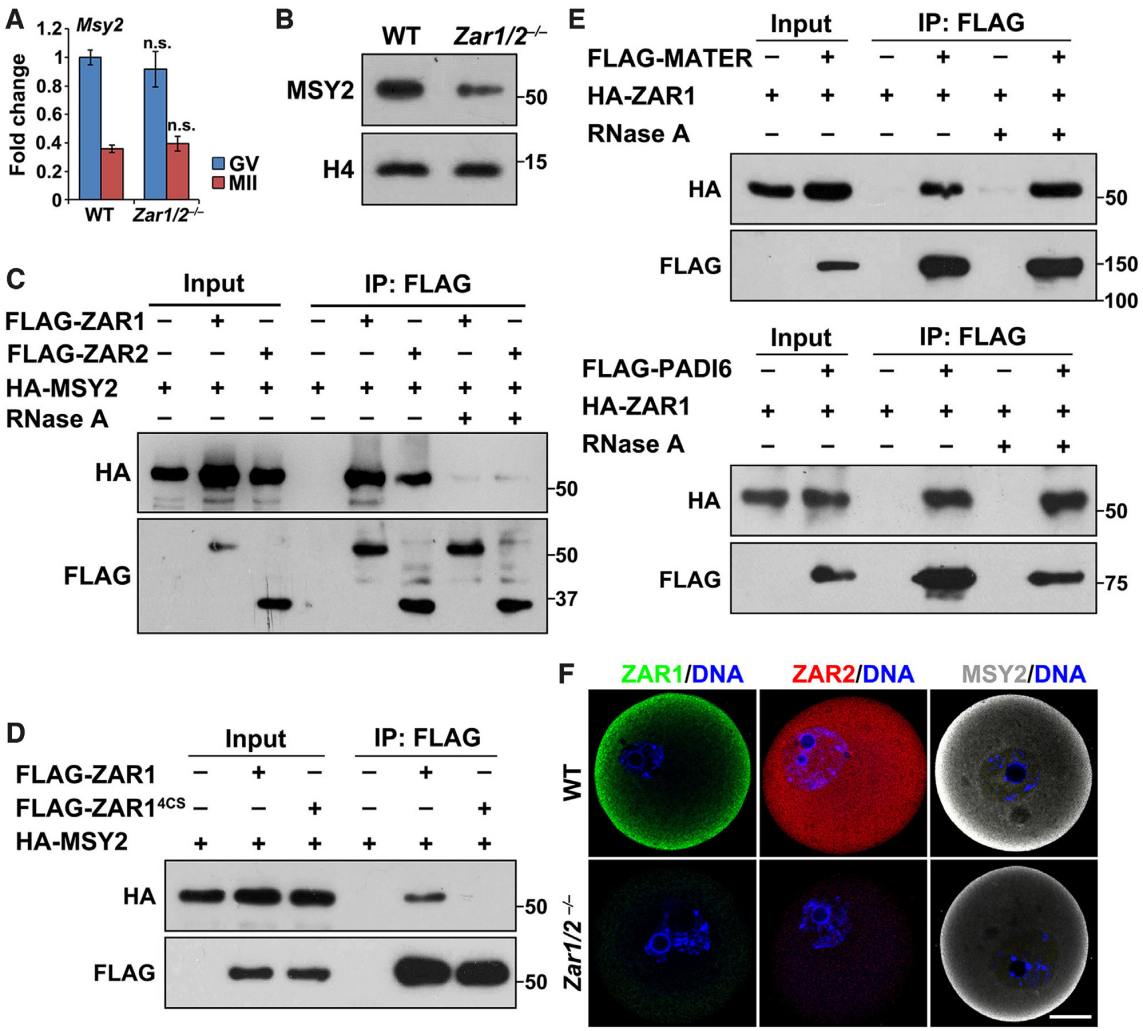
ZAR1/2 interact with MSY2 at CPLs. (**A**) RT-qPCR results showing *Msy2* mRNA levels in the oocytes of WT and *Zar1/2^–/–^* females. *n* = 3 biological replicates. Error bars, S.E.M. n.s.: non-significant. (**B**) Western blot results showing MSY2 expression in WT and *Zar1/2^–/–^* oocytes at the GV stage. Total proteins from 50 oocytes were loaded in each lane. Histone H4 was blotted as a loading control. (**C**) Co-IP results showing the interaction between MSY2 and ZAR1/2. Experiments in (C**–E**) were performed three times with reproducible results; a representative result is shown. (D) Co-IP results showing the interactions of MSY2 with WT and zinc finger-mutated ZAR1 (ZAR1^4CS^). (E) Co-IP results showing the interactions of ZAR1 with CPL components MATER (top) and PADI6 (bottom). (**F**) Immunofluorescent staining of ZAR1/2 and MSY2 in WT and *Zar1/2^–/–^* oocytes. Scale bar, 20 μm.

In addition to oocyte maturation defects, we observed that the levels of maternal mRNAs decreased in GV-stage arrested *Zar1/2* knockout oocytes (Figure 3A). Therefore we further investigated the possible causes for the maternal transcriptome change. MSY2 is an RNA-binding protein that maintains global mRNA stability in mammalian germ cells (46,47). *Zar1/2* knockout caused phenotypes resembling *Msy2* knockout; female *Msy2^–/–^* mice are infertile as their MII oocytes are severely compromised due to aberrant spindle formation and chromosome alignment (48). Therefore, we examined potential functional connections between ZAR1/2 and MSY2, finding that MSY2 protein levels, but not mRNA levels, decreased in *Zar1/2^–/–^* oocytes (Figure 7A and B). When co-expressed in HeLa cells, MSY2 coprecipitated with ZAR1/2 but their interaction was disrupted by RNase A treatment (Figure 7C), indicating that ZAR1/2 and MSY2 do not directly bind to each other but reside on the same RNA molecules. Furthermore, the non-RNA binding ZAR1 mutant (ZAR1^4CS^) did not coprecipitate with MSY2 (Figure 7D), again suggesting that the MSY2–ZAR1/2 interaction is indirect and RNA-mediated.

PADI6 anchors MSY2-mRNAs to oocyte cytoplasmic lattices (CPLs), which have a function in mRNA and ribosomes storage during MZT (46,49–50). CPLs contain several proteins encoded by *Mater, Padi6, Floped, Tle6* and *Filia*, whose deficiency in oocytes cause CPL disassembly, abnormal subcellular structure clustering (mitochondria and Golgi apparatus) and zygotic arrest (51). ZAR1/2 could bind the CPL components MATER and PADI6 when co-expressed in HeLa cells. These interactions were resistant to RNase A digestion, suggesting that they are not RNA-mediated (Figure 7E and Supplementary Figure S7D).

Immunofluorescence showed that ZAR1 and MSY2 were both enriched in the cortical region of fully grown oocytes, whereas ZAR2 was distributed evenly in the oocytes (Figure 7F). ZAR1/2 fluorescent signals were shown to be specific since they were not detected in *Zar1/2^–/–^* oocytes under the same labeling and imaging conditions. Consistent with western blotting, the MSY2 immunofluorescent signal decreased in *Zar1/2^–/–^* oocytes; however, its cortical enrichment persisted. Collectively, these results suggest that ZAR1/2 may function together with MSY2 and CPL, which have been reported to be involved in maternal mRNA stabilization and mobilization in oocytes (46–47,49).

## DISCUSSION

**Figure 8.**
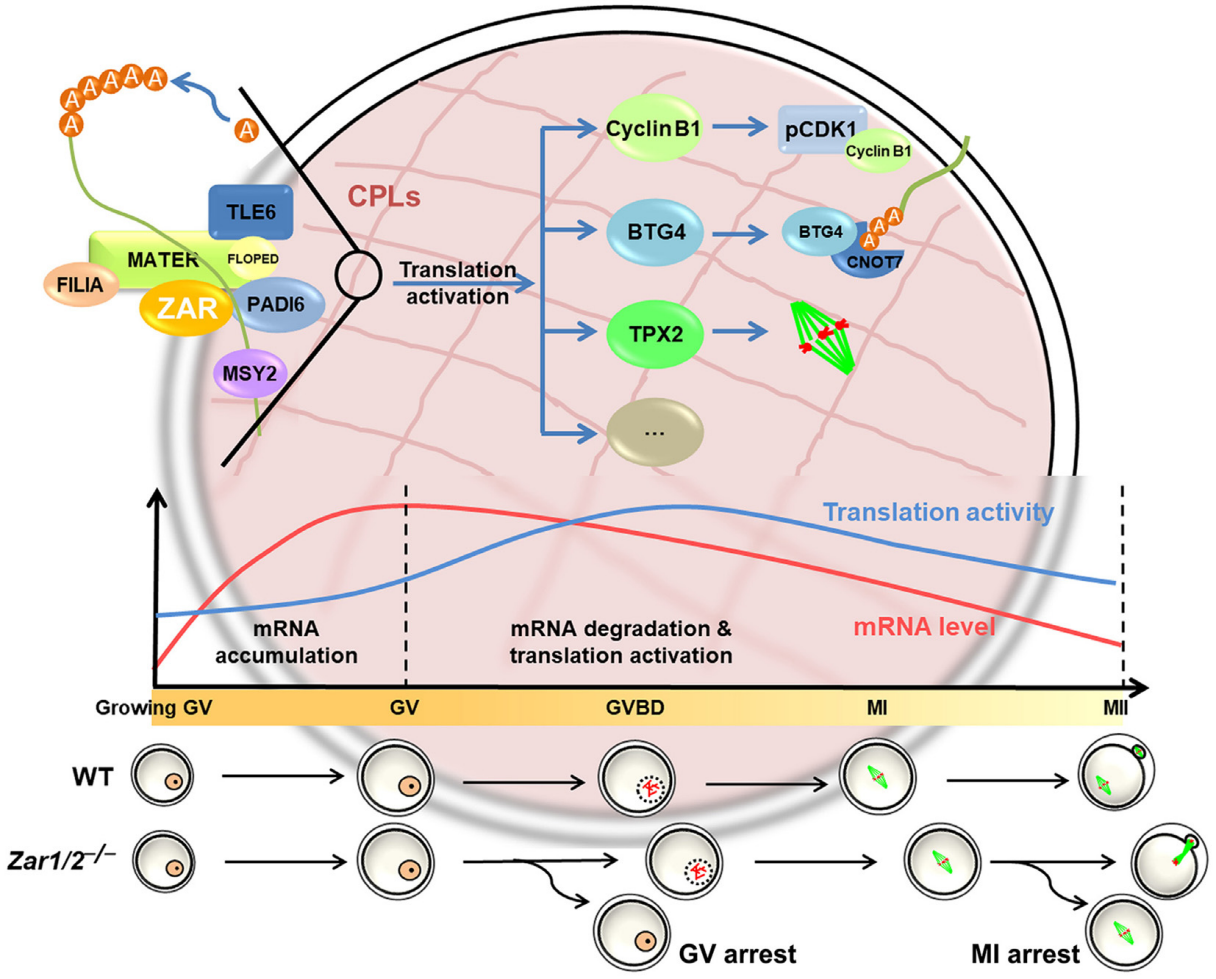
A diagram showing the role of ZAR1/2 in the regulation of mRNA stability and translation in oocytes. In GV-arrested oocytes, ZAR1 and ZAR2 are involved in the maintenance of mRNA stability together with MSY2 at the CPLs. After meiotic resumption of the oocytes, ZAR1/2 facilitate the translation activation of certain maternal mRNAs, including *Ccnb1*, *Tpx2* and *Btg4*, which encode proteins involved in cell-cycle progression, spindle assembly and maternal mRNA decay. ZAR1/2 deletion in oocytes results in MSY2 downregulation, maternal mRNA instability, the impaired translation of important oocyte proteins and ultimately meiotic maturation defects.

*Zar1* was one of the first reported mammalian maternal effect genes together with *Mater* and *Npm2* (19–21). Wu *et al.* reported that *Zar1*-null oocytes displayed normal meiotic maturation but zygotes derived from these oocytes arrested at the 1-cell stage (17,18). Although the phenotype of *Zar1* knockout mice was reported in 2002, the mechanisms underlying the regulation of MZT by mammalian ZAR1 have not been investigated. Additionally, *Zar2* was reported to be a *Zar1* homolog in the vertebrate genome, yet its *in vivo* functions remain unknown (25,26). In this study, we generated *Zar1* and *Zar2* knockout mice using the CRISPR-Cas9 system, demonstrated their novel function regulating oocyte meiotic maturation, and investigated the biochemical mechanisms that caused their phenotypes (Summarized in Figure 8). These new results suggest that: (i) the zygotic arrest phenotype in *Zar1/2*-null mice is secondary to meiotic maturation failure in the *Zar1/2^–/–^* oocytes; and (ii) *Zar2* is partially redundant with *Zar1* in regulating oocyte maturation and MZT since maternal *Zar1/2* knockout causes stronger oocyte and zygote development defects than *Zar1*-deletion alone.

*Zar1* mutations in zebrafish cause oocyte protein over-translation and induce protein folding stress in ooplasm (22). Consequently, growing oocytes undergo P53-dependent apoptosis and female germ cells are depleted in female gonads. By comparison, *Zar1/2* deletion in mice cause no morphological abnormalities during intra-ovarian oocyte development or histological changes in the gonad. Contrary to the reports in zebrafish, translational activity decreased in *Zar1/2*-null mouse oocytes, particularly after meiotic resumption. Nevertheless, these studies all indicate that ZAR1 primarily functions during oocyte growth (zebrafish) or maturation (mouse), rather than after fertilization as previously believed. The role of ZAR1 in oocyte maturation had not been noted in previous studies (17), potentially because ZAR2 can partially substitute for ZAR1 in maturing oocytes. Only by deleting both *Zar1* and *Zar2* can we clearly observe GVBD, PB1 emission and spindle defects.

Biochemical studies in *Xenopus* oocytes have shown that ZAR1/2 interact with mRNA 3′-UTRs to repress translation, contrary to the translation-stimulating activity we observed in mouse oocytes (28). This may be due to the following reasons: (i) while the role of *Xenopus* ZAR1/2 was mainly investigated in immature GV stage-arrested oocytes, we observed that ZAR1/2 facilitates maternal mRNA translation most significantly after meiotic resumption; and (ii) the translational inhibition effect of *Xenopus* ZAR1/2 was tested using a β-globin 3′-UTR-based reporter containing two MS2 stem-loops, which are recognition motifs of the RNA-binding protein Musashi. Instead, we evaluated the influence of ZAR1/2 on translation using the 3-UTRs of bona fide ZAR1/2-targeting mRNAs during oocyte meiotic resumption, including *Btg4*, *Ccnb1* and *Wee2*. The results of the reporter assay were consistent with the observation that endogenous proteins encoded by these mRNAs failed to accumulate in *Zar1/2*-null oocytes. Among the proteins transiently translated from maternal mRNAs, cyclin B1 and WEE2 are associated with the timely progression and arrest of the meiotic cell cycle (40); TPX2 regulates spindle assembly and polar body emission (44,45), while BTG4 triggers maternal mRNA clearance and MZT (10,39). The translation of these important oocyte proteins was compromised in the absence of ZAR1/2 and their functions correspond with the major phenotypes of *Zar1/2*-null oocytes.

In addition to oocyte maturation defects, we observed transcriptomic changes in GV oocytes after *Zar1/2* knockout, with the levels of many maternal mRNAs decreasing in GV oocytes. This is unlikely to be due to decreased transcription as: (i) total transcriptional activity in growing *Zar1/2*-null oocytes was comparable to that in WT oocytes; and (ii) ZAR1/2 are cytoplasmic proteins that lack biochemical bases to directly regulate transcription. Therefore, a plausible explanation is that ZAR1/2 may be involved in the stabilization of mRNAs in the ooplasm. Mammalian oocytes accumulate large quantities of translationally dormant mRNAs during their growth phase, with oocyte maturation depending on the timely recruitment of maternal mRNAs to translational machinery (4,52). MSY2 is an RNA-binding protein expressed abundantly in mouse ooplasm that plays a key role in stabilizing maternal mRNAs in oocytes (53). A specialized cytoskeletal structure known as the CPL is important for compartmentalizing the ooplasm and mRNA storage in oocytes (50,51). ZAR1 indirectly interacted with MSY2 via RNAs, directly bound CPL components, and colocalized with MSY2 and CPL in the cortical region of GV oocytes. Furthermore, MSY2 expression was low in *Zar1/2* null oocytes, possibly due to the decreased stability and translation of *Msy2* mRNAs, as for other maternal transcripts.

These results reveal a previously unrecognized function of ZAR1/2 in mammalian oocytes and indicate that the previously described role of ZAR1/2 in ZGA is secondary to oocyte maturation defects. The stringent definition of a maternal factor is ‘a protein supplied by the mother but solely functioning in the progeny’ (54,55); therefore, a true maternal effect gene determines the phenotype of the offspring but not the mother itself. In this sense, *Zar1*, known for decades as a representative mammalian maternal effect gene, is actually not a true maternal effect gene.

## DATA AVAILABILITY

RNA-seq data have been deposited in the NCBI Gene Expression Omnibus database. GEO accession number: GSE135787. UCSC genome browser session for RNA-seq data: http://genome.ucsc.edu/s/yezhang_zhu/Zar1%262

## Supplementary Material

gkz863_Supplemental_Files
