# Supplementary material for: ZAR1 and ZAR2 are required for oocyte meiotic maturation by regulating the maternal transcriptome and mRNA translational activation
Source: Nucleic Acids Res. 2019 Oct 10;47(21):11387–402. doi: 10.1093/nar/gkz863 (PMC6868374; doi:10.1093/nar/gkz863)
Supplement: gkz863_Supplemental_Files [file gkz863_supplemental_files.zip › Zar1 and Zar2 paper Supplementary.pdf]

Supplementary Information

Supplementary Figures

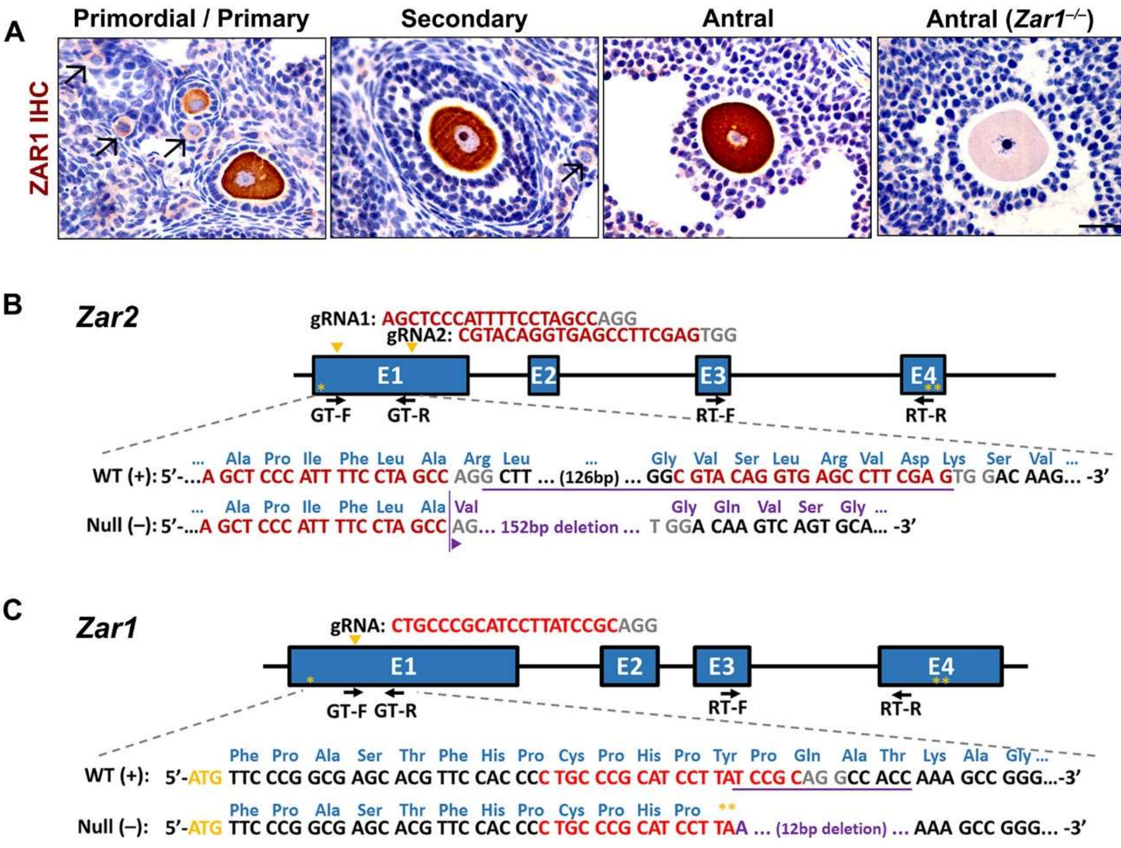

Figure S1. Expression of ZAR1 in oocytes and the generation of *Zar1*$^{-/-}$ and *Zar2*$^{-/-}$ mice. **A:** Immunohistochemistry results showing ZAR1 protein levels in the oocytes of WT and *Zar1/2*-null mice. Follicle stages are indicated. Arrows indicate oocytes within primordial follicles. Scale bar, 50 µm. **B-C:** Gene-targeting strategies for *Zar2* (**B**) and *Zar1* (**C**) knockout. F, forward; R, reverse; gRNA, guide RNA; *, start codon; **, stop codon.

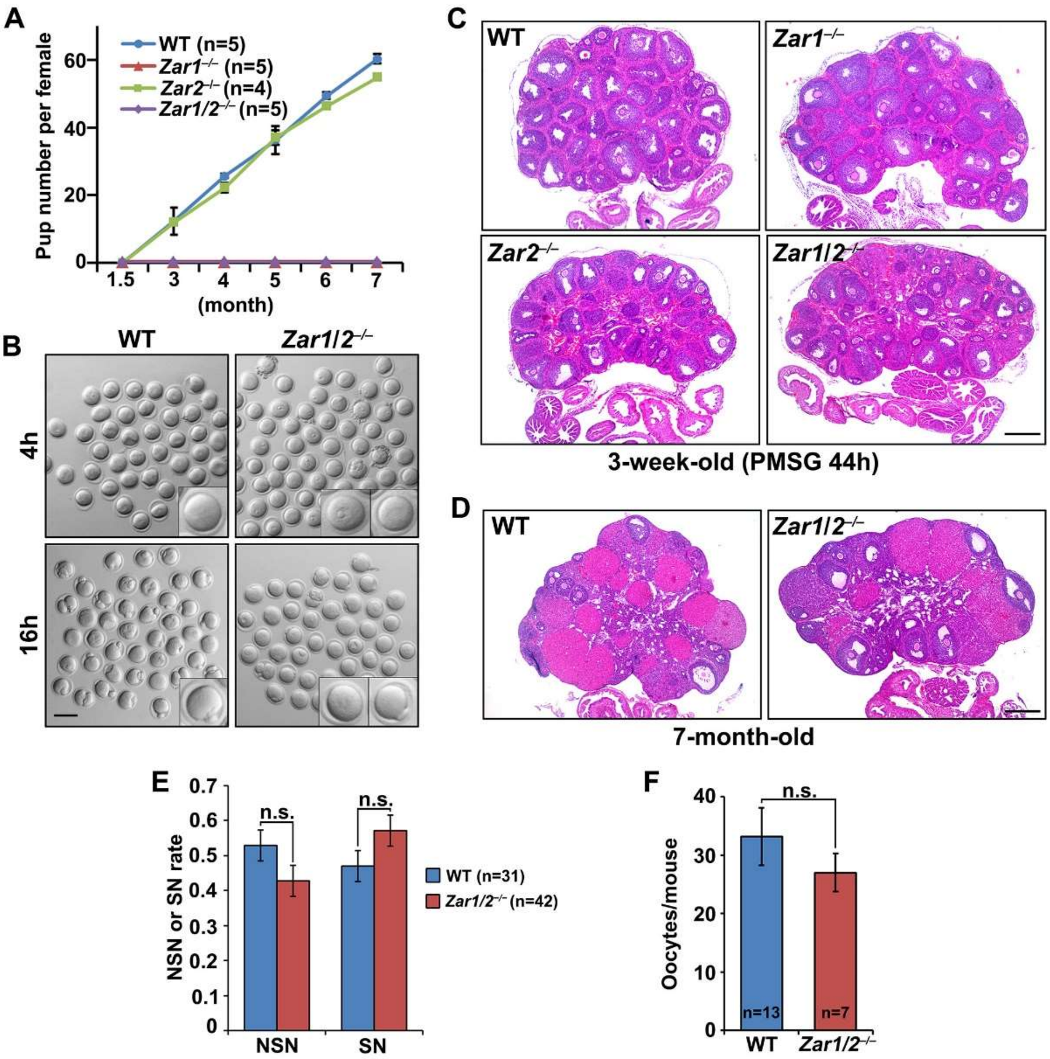

Figure S2. Phenotype analyses of female *Zar1*$^{-/-}$, *Zar2*$^{-/-}$, and *Zar1/2*$^{-/-}$ mice. A: Cumulative number of pups per female showing the fertility of female WT, *Zar1*$^{-/-}$, *Zar2*$^{-/-}$, and *Zar1/2*$^{-/-}$mice. The number of analyzed mice is indicated (n). Error bars, S.E.M. **B:** Representative images of WT and *Zar1/2*$^{-/-}$ oocytes cultured *in vitro*. Scale bar, 100 µm. **C:** H&E staining results showing the ovarian histology of 3-week-old WT and *Zar1/2*$^{-/-}$ mice 44 h after PMSG injection. Scale bar, 500 µm. **D:** H&E staining results showing the ovarian histology of 7-month-old WT and *Zar1/2*$^{-/-}$ mice. Scale bar, 500 µm. **E:** Proportion of non-surrounded nucleolus (NSN)-type and surrounded nucleolus (SN)-type fully-grown GV oocytes from WT and *Zar1/2*$^{-/-}$ females. **F:** The average number of ovulated oocytes in 3-week-old WT and *Zar1/2*$^{-/-}$ females, as determined by a superovulation assay. In (**E**) and (**F**), the numbers of analyzed oocytes and mice are indicated (n), respectively. Error bars, S.E.M. n.s.: non-significant.

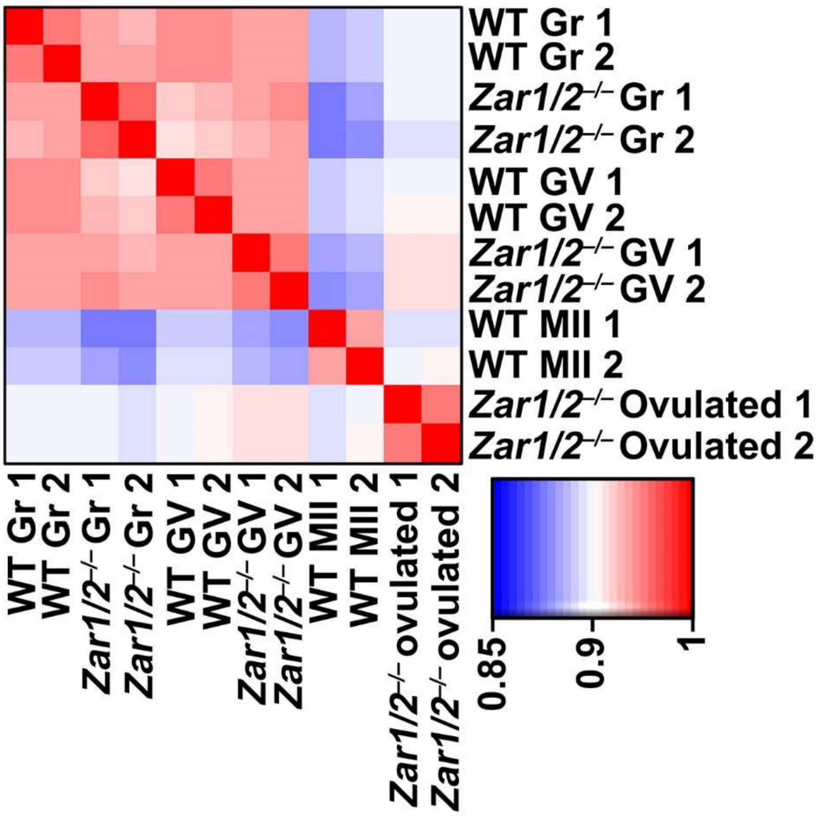

Figure S3: Heat map of the Spearman correlation coefficients of WT and *Zar1/2*$^{-/-}$ oocytes at the indicated developmental stages (Gr: growing stage).

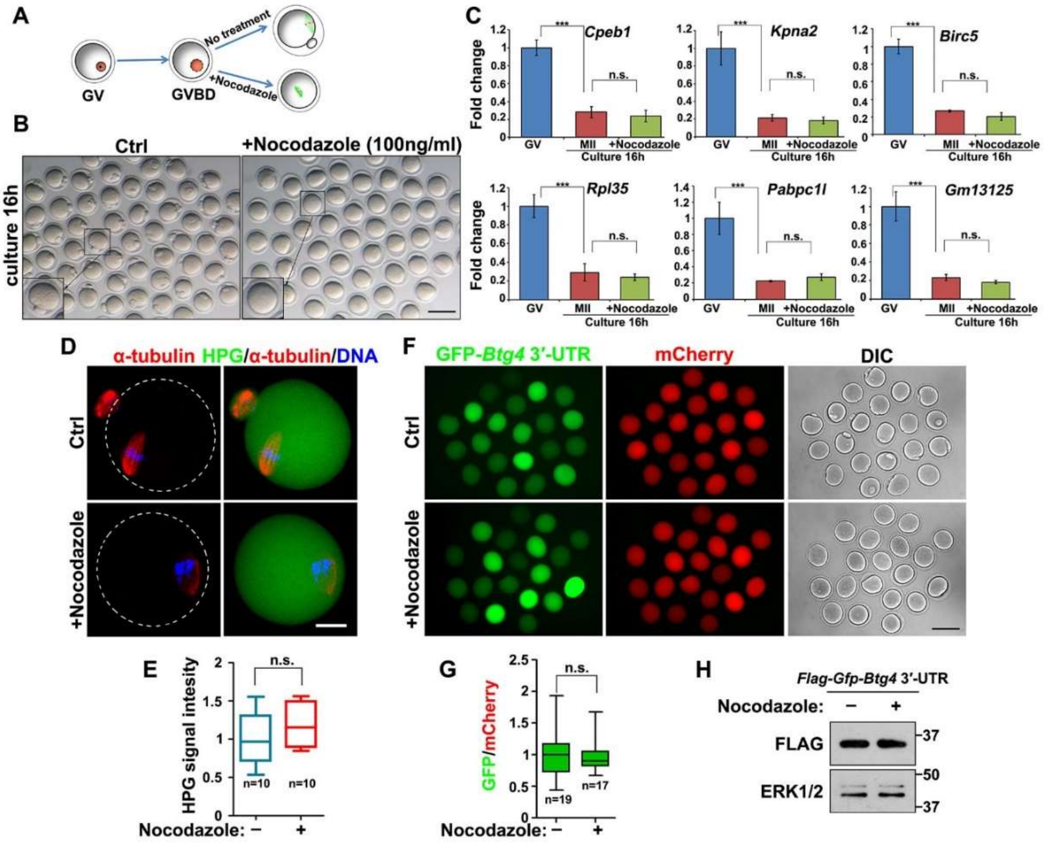

Figure S4. Impaired spindle assembly and PB1 emission in oocytes do not affect the degradation or translational activation of maternal mRNAs. A: Diagram of spindle assembly disruption using the microtubule disruptor, nocodazole. **B:** Representative images of oocytes with or without nocodazole treatment 16 h after *in vitro* culture. Scale bar, 100 μm. **C:** Quantitative RT–PCR results showing the relative levels of indicated transcripts in oocytes with or without nocodazole treatment 16 h after *in vitro* culture. n = 3 biological replicates. Error bars, SEM. $***P < 0.001$ by two-tailed Student's *t*-tests. n.s.: non-significant. **D-E:** HPG fluorescent staining **(D)** and quantification of HPG signal intensity **(E)** showing protein synthesis in oocytes with or without nocodazole treatment 16 h after *in vitro* culture. Scale bars, 20 μm. The number of analyzed oocytes for each treatment is indicated (n). Error bars, SEM. n.s.: non-significant. **F-H:** Fluorescence microscopy **(F)**, relative GFP intensity relative to mCherry by fluorescence microscopy **(G)** and western blot analysis **(H)** showing the expression of FLAG-GFP fused with *Btg4* 3′-UTR in oocytes with or without nocodazole treatment 16 h after *in vitro* culture. The number of analyzed oocytes is indicated (n). Error bars, S.E.M. n.s.: non-significant. Total proteins from 100 oocytes were loaded in each lane. ERK1/2 were blotted as a loading control.

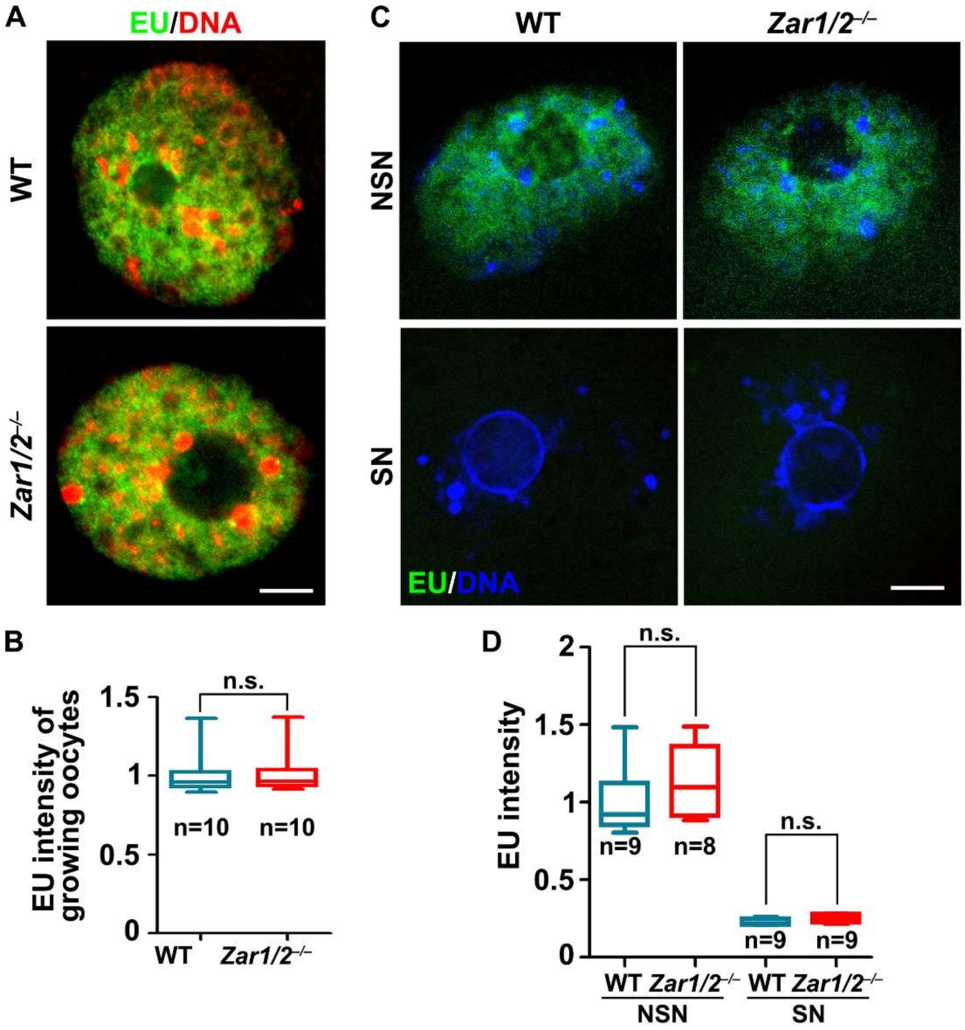

Figure S5. ***Zar1/2*** **deletion impaired the translational activation but not the transcription of maternal mRNAs. A-B:** Results of EU fluorescent staining **(A)** and quantification of EU signal intensity **(B)** showing the transcriptional activity of mRNA in growing oocytes from WT and *Zar1/2*$^{-/-}$ females. Oocytes were incubated in M16 medium with 1 mM EU for 1 h prior to staining. Scale bars, 5 μm. The number of analyzed oocytes is indicated (n). Error bars, SEM. n.s.: non-significant. **C-D:** EU staining **(C)** and quantification of EU **(D)** signal intensity results showing the transcriptional activity of mRNA in GV oocytes with NSN or SN nucleolar configurations from WT and *Zar1/2*$^{-/-}$ females. Scale bars, 5 μm. The number of analyzed oocytes is indicated (n). Error bars, SEM. n.s.: non-significant.

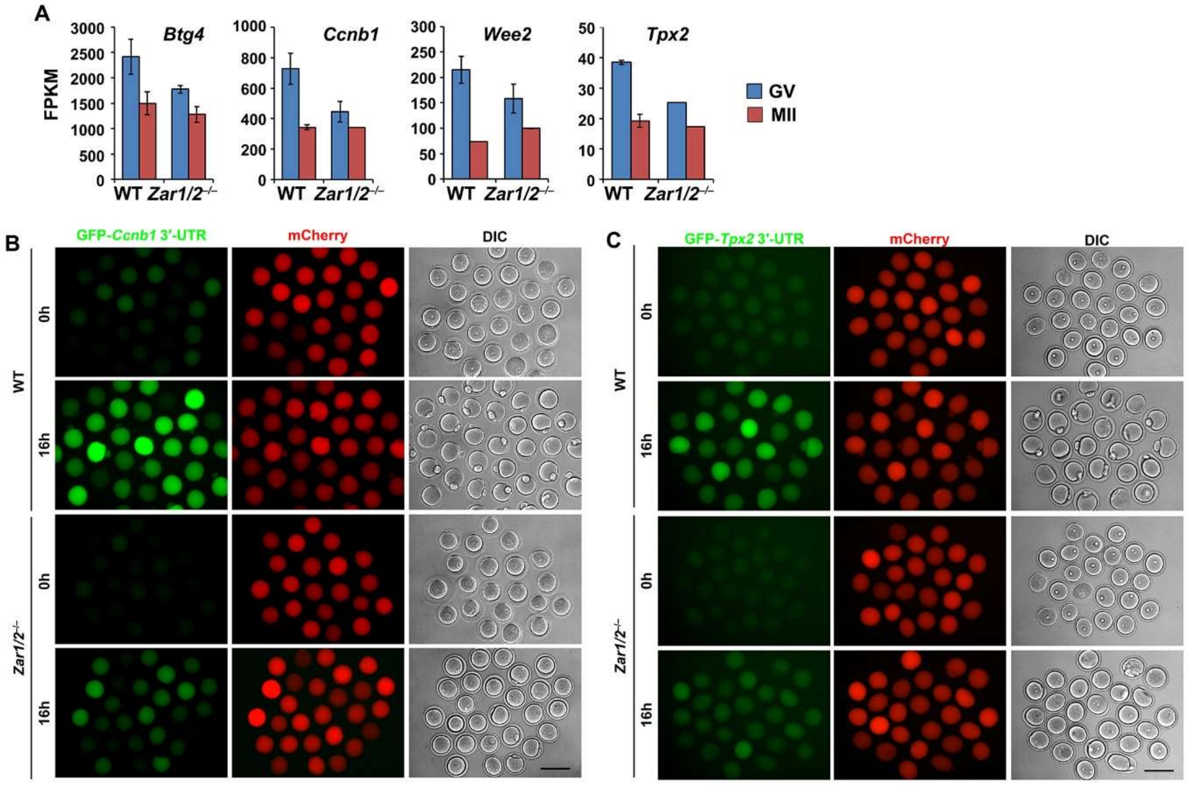

Figure S6. ZAR1/2 regulate the translational activation of mRNAs via their 3′-UTR. A: FPKMs of indicated genes extracting from RNA-seq results showing their expression levels in WT and *Zar1/2*$^{-/-}$ oocytes. Error bars, S.E.M. **B-C:** Fluorescence microscopy showing the expression of FLAG-GFP fused with *Ccnb1* **(B)** and *Tpx2* **(C)** 3′-UTRs in WT and *Zar1/2*$^{-/-}$ oocytes. Scale bar, 100 μm.

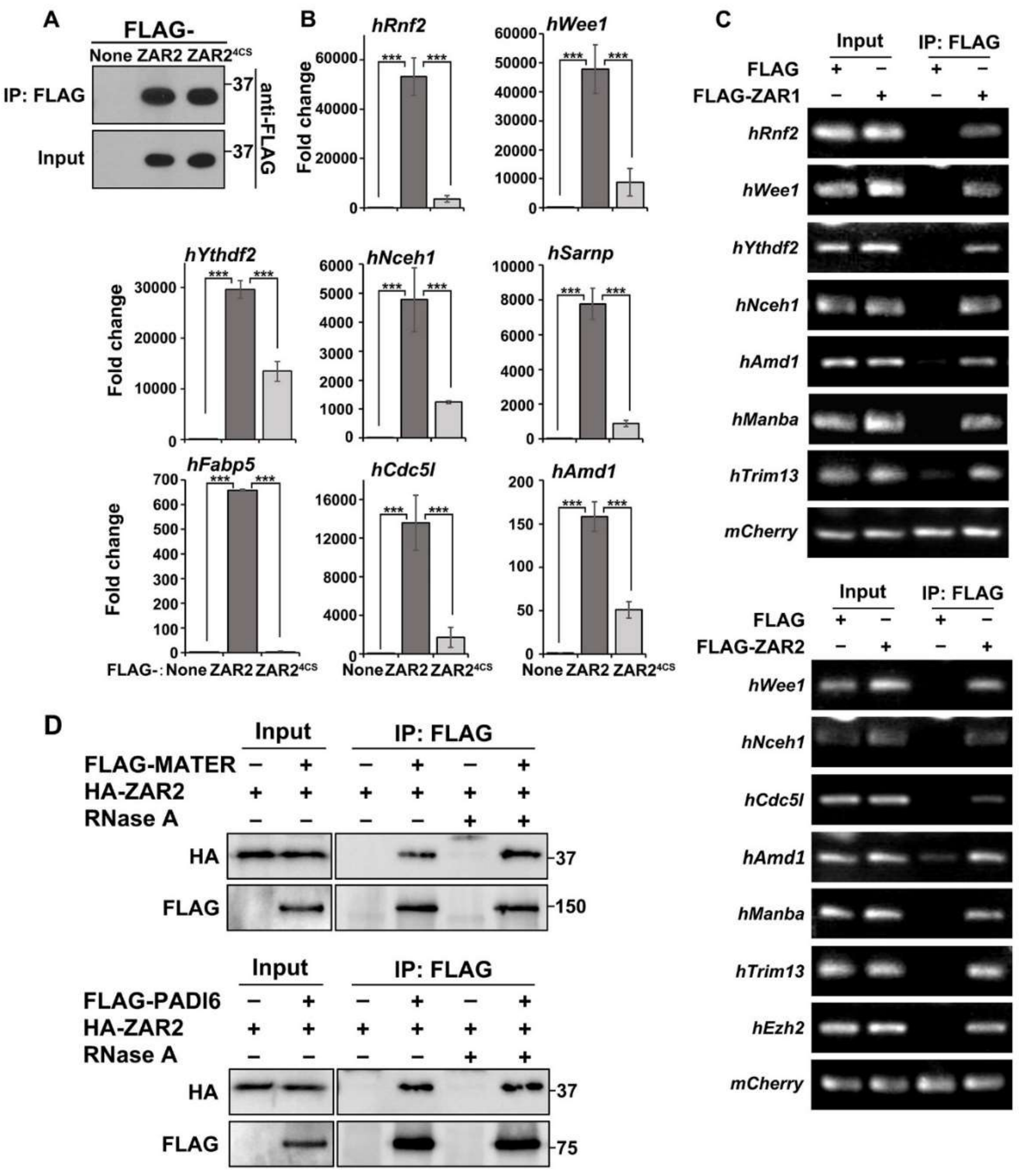

Figure S7. ZAR1/2 bind mRNAs via four conserved cysteines. A: Western blot results showing that ZAR2 (Flag-ZAR2 and Flag-ZAR2^{4CS}) was specifically pulled down from HeLa cell lysates using an anti-Flag antibody. **B:** RIP assay results using an anti-Flag antibody showing the interactions between ZAR2 (Flag-ZAR2 and Flag-ZAR2^{4CS}) and the indicated transcripts in HeLa cells. The levels of the mRNAs coprecipitated with ZAR2 were detected by RT-qPCR. n = 3 biological replicates. Error bars, S.E.M. ***$P < 0.001$ by two-tailed Student's *t*-tests. **C:** RT-PCR results showing the interactions between ZAR1/2 and the indicated transcripts in HeLa cells. *In vitro* transcribed *mCherry* mRNAs were added to all samples before reverse transcription as a loading control. **D:** Co-IP results showing the interactions of ZAR2 with CPL components MATER (top) and PADI6 (bottom).

Supplementary Tables

Table S1. Primer sequences.

Primer name	Target Gene	Application	Sequences (5′-3′)
Zar1 WT-F	*Zar1*	Genotyping of WT allele	5′-GCTCGGCTAATCTCGGTGATGA-3′
Zar1 WT-R			5′-GCTTTGGTGGCCTGCGGA-3′
Zar1 KO-F		Genotyping of KO allele	5′-TCGGCTAATCTCGGTGATGATT-3′
Zar1 KO-R			5′-CAGCCATCCCCGGCTTTATA-3′
Zar1-F		Real-time PCR	5′-AGAGCGCCTATGTGTGGTGT-3′
Zar1-R			5′-TCTCCCACACAAGTCTTGCC-3′
Zar2 GT-F	*Zar2*	Genotyping	5′-GCTCTGGAATAAATTAAGAAACCTG-3′
Zar2 GT-R			5′-TCCTGGCTTTCCTCGTCTTC-3′
Zar2-F		Real-time PCR	5′-TATTTCAAACAGCTCTGTAACAAGTGCC-3′
Zar2-R			5′-AGGAGAATTTCTTGTCTTTGCAGTGG-3′
Zp2-F	*Zp2*	Real-time PCR	5′-GCAGCTGGAGCTCTTGTTCT-3′
Zp2-R			5′-TCCATTGTCCAAAGTCCACA-3′
Tceb1-F	*Tceb1*	Real-time PCR	5′-ATCTTCTGATGGCCATGAATTT-3′
Tceb1-R			5′-CCTTGTAGGTAAAATACATGCACAC-3′
Birc5-F	*Birc5*	Real-time PCR	5′-GAGGCTGGCTTCATCCACTG-3′
Birc5-R			5′-CTTTTTGCTTGTTGTTGGTCTCC-3′
Padi6-F	*Padi6*	Real-time PCR	5′- AGTGTATCAGCCTGAACCGC-3′
Padi6-R			5′- AGGTGCCATTGATTTTGGG-3′
Npl-F	*Npl*	Real-time PCR	5′-GAACAGAAGGACTTGGCTTCAGC-3′
Npl-R			5′-CCTCAGCCTTAGCAGTAAACTCCTG-3′
Zscan4d-F	*Zscan4d*	Real-time PCR	5′- CAATGCAAGGACAAGAAGCTCTCTT-3′
Zscan4d-R			5′- CTGGCATCAAGAGGGAATTGAAA-3′
Zscan4f-F	*Zscan4f*	Real-time PCR	5′- CAACACCAGATAATGAGCAGATGCC-3′
Zscan4f-R			5′- CGGCAGTAGTCGGAGCACTCG-3′
Zscan5b-F	*Zscan5b*	Real-time PCR	5′- ATGGGCAATACAGAAGATGGGC-3′

Zscan5b-R			5′- GGTCAAACCGGGACTTGTAAA-3′
Dppa4-F	*Dppa4*	Real-time PCR	5′-AGTCAACCTAGCACGGCTC-3′
Dppa4-R			5′-TCCTGGCGTCTCAGTGTCT-3′
Ppp1r8-F	*Ppp1r8*	Real-time PCR	5′-CCACAACAAGCGGATTTCAACC-3′
Ppp1r8-R			5′-CCACTGAAGGCAAAGTTCTGCATG-3′
Polr2j-F	*Polr2j*	Real-time PCR	5′- GCCTTCGAGTCGTTCTTGCT -3′
Polr2j-R			5′- GCGATTTAATGATGTTCCCCAGA -3′
MuERV-L-F	*MuERV-L*	Real-time PCR	5′-ATCTCCTGGCACCTGGTATG-3′
MuERV-L-R			5′-AGAAGAAGGCATTTGCCAGA-3′
Eif1a-F	*Eif1a*	Real-time PCR	5′-AACAGGCGCAGAGGTAAAAA-3′
Eif1a-R			5′-GCACAGCCTCCTTACACCAT-3′
Tho4-F	*Tho4*	Real-time PCR	5′-CGAGGCACTGGGTCTAAGAG-3′
Tho4-R			5′-CCAATGAACAGGTCATGCTG-3′
Actin-F	*Actin*	Real-time PCR	5′- GCTCTTTTCCAGCCTTCCTT -3′
Actin-R			5′- GTACTTGCGCTCAGGAGGAG -3′
Nceh1-F	*Nceh1*	Real-time PCR	5′-CATCCGTGCCACTAAATATTTC-3′
Nceh1-R			5′-GTGTGTTGAAGTCCAAAGCCT-3′
Btg4-F	*Btg4*	Real-time PCR	5′-TGAAAAAGCATGAGAAACTGAGTAC-3′
Btg4-R			5′-CCCATCTACCTTTAAAAGAAGCAA-3′
Paip2-F	*Paip2*	Real-time PCR	5′-GAACGCTGTTTCCAAGAAATGC-3′
Paip2-R			5′-CCAGAGAAGAGCCATCACTGATA-3′
Nobox-F	*Nobox*	Real-time PCR	5′-CATGAAGGGGACCTGAAGAA-3′
Nobox-R			5′-GGAAATCTCATGGCGTTTGT-3′
Wee2-F	*Wee2*	Real-time PCR	5′-ACCCAGCTCCAAAGGAGAGA-3′
Wee2-R			5′-TCTGAGGGGTCTGGATATGC-3′
Rnf2-F	*Rnf2*	Real-time PCR	5′-GAGTTACAACGAACACCTCAGG-3′
Rnf2-R			5′-CAATCCGCGCAAAACCGATG-3′
Cnot7-F	*Cnot7*	Real-time PCR	5′-GGTGGATTACAGGAAGTTGCTG-3′
Cnot7-R			5′-GGATGAGCCAGAACCAAGG-3′

Msy2-F	*Msy2*	Real-time PCR	5′-CAACCAGCAACAGCCCATAGAGG-3′
Msy2-R			5′-TTGGCTGGGCTTGGTCTCTCC-3′
Cdc5l-F	*Cdc5l*	Real-time PCR	5′-GTGAAGCTTACAACCAGGTGTG-3′
Cdc5l-R			5′-GCAGCTCTCTTGGCTTCTG-3′
Ythdf2-F	*Ythdf2*	Real-time PCR	5′-GAGAATAAACCAGTGACCAACTCT-3′
Ythdf2-R			5′-GCTTTCTATTTCCCACGACCT-3′
Ccnb1-F	*Ccnb1*	Real-time PCR	5′-AAGGTGCCTGTGTGTGAACC-3′
Ccnb1-R			5′-GTCAGCCCCATCATCTGCG-3′
Tpx2-F	*Tpx2*	Real-time PCR	5′-GATGCCCCCACCGACTTTATC-3′
Tpx2-R			5′-CTTGTTCTCCAAGTTGGCCTT-3′
18S-F	18S rRNA	Real-time PCR	5′-TGATTAAGTCCCTGCCCTTTG-3′
18S-R			5′-CTTCTCTCACCTCACTCCAGACAC-3′
28S-F	28S rRNA	Real-time PCR	5′-CGTGTGAGTAAGATCCTCCACC-3′
28S-R			5′-GAGTTTACCACCCGCTTTGG-3′
Gfp-F	*Gfp*	Real-time PCR	5′-CGCTACCCCGACCACATGAA-3′
Gfp-R			5′-CTTCAGCTCGATGCGGTTCA-3′
hRnf2-F	*hRnf2*	Real-time PCR	5′-GCCACTGTTGATCACTTATCCA-3′
hRnf2-R			5′-TCCATGGGTTTGTTCACTTTC-3′
hWee1-F	*hWee1*	Real-time PCR	5′-GAAGACCTTCAGCAATGGCA-3′
hWee1-R			5′-AGTGAAGAGTGCTCTTTCCTCA-3′
hYthdf2-F	*hYthdf2*	Real-time PCR	5′-ATGAAGTCTGTTGTGGACTATAATGC-3′
hYthdf2-R			5′-GCTTAGCTTTTTCTAGGGGTACC-3′
hNceh1-F	*hNceh1*	Real-time PCR	5′-ACAAAGTATTTCCTGAAGCCAGA-3′
hNceh1-R			5′-CTGGATAAATTAAAGCTTGTAGTTTG-3′
hSarnp-F	*hSarnp*	Real-time PCR	5′-TGAATGTCTCTTCAATCTCCAGAA-3′
hSarnp-R			5′-CAGGAACTTTTCATCAGGCA-3′
hFabp5-F	*hFabp5*	Real-time PCR	5′-CTCAGACTGTCTGCAACTTTACAGATGG-3′
hFabp5-R			5′-AGATTTGCTCATTGAACTGAGCTTGG-3′
hAmd1-F	*hAmd1*	Real-time PCR	5′-TATTCACATCACTCCAGAACCAG-3′

hAmd1-R			5′-CAATCAAGACGCTTAAAACCTTC-3′
hCdc5l-F	*hCdc5l*	Real-time PCR	5′-GTGCTATGGGGCTCATGAA-3′
hCdc5l-R			5′-CCAGCAGCAAATCAGCATATC-3′
hManba-F	*hManba*	Real-time PCR	5′-TGGTTTCCTTTTACCTTTCAGC-3′
hManba-R			5′-GCTTCCTACATCCAACCAAAC-3′
hTrim13-F	*hTrim13*	Real-time PCR	5′-GCTTTGCCCTGGGAAATAGTAAC-3′
hTrim13-R			5′-ATTCCAGTAGCTGAAGTTTCCTTACG-3′
hEzh2-F	*hEzh2*	Real-time PCR	5′-ATGAATTCATCTCAGAATACTGTGG-3′
hEzh2-R			5′-CTTTTGCATAGCAGTTTGGATT-3′
mCherry-F	*mCherry*	Real-time PCR	5′-CCGTAATGCAGAAGAAGACCATG-3′
mCherry-R			5′-GCGTTCGTACTGTTCCACGATG-3′
Cpeb1-F	*Cpeb1*	Real-time PCR	5′-GGACCTTCTTGGAGCTCCTA-3′
Cpeb1-R			5′-GAACGCCTCCTAGGAACACC-3′
Kpna2-F	*Kpna2*	Real-time PCR	5′-ATGTCCACGAACGAGAATGCT-3′
Kpna2-R			5′-AAGGAGCTGACGTTTCTTCTTTT-3′
Rpl35-F	*Rpl35*	Real-time PCR	5′-GAGCTGTTGAAACAACTGGACG-3′
Rpl35-R			5′-CGAACGACTCGTATCTTGGAGAG-3′
Pabpc1l-F	*Pabpc1l*	Real-time PCR	5′-GCTTCTCGTGCCACATAGAGGAAC-3′
Pabpc1l -R			5′-AGCTCCAGGTTGTCCAGCTCC-3′
Gm13125-F	*Gm13125*	Real-time PCR	5′-GTTACCCAAGACCACTCAAACA-3′
Gm13125-R			5′-CCACTGAACGAGGTAGGAGAA-3′
R1	(Poly A)	Anchor primer for PAT assay	5′-GCGAGCTCCGCGGCCGCGTTTTTTTTTTTT-3′
Btg4 PAT-F	*Btg4*	PAT assay	5′-GTAGGTTTTCAACTAAGGAAGAT-3′
Ccnb1 PAT-F	*Ccnb1*	PAT assay	5′-CCGTGACAAAGGCATAACTC-3′
Wee2 PAT-F	*Wee2*	PAT assay	5′-GACTATCATTGTGTGAAACAACTAGAGTAAGAC-3′
Tpx2 PAT-F	*Tpx2*	PAT assay	5′-CTGGAAGTCAGTCTTCCACTAG-3′
Gapdh PAT-F	*Gapdh*	PAT assay	5′-ACTGAGCAAGAGAGGCCCTA-3′

h: means human.

Table S2. Antibody information.

**Protein name**	**Manufacture (catalogue number)**	**Applications (working dilution)**
ZAR1	Made by Abcam company	IF (1:1000); WB (1:3000); IP (1:1000); IHC (1:500)
ZAR2	Made by company	IF (1:1000); WB (1:5000)
DDB1	Epitomics (3821-1)	WB (1:10000)
FITC-α-tubulin	Sigma (F2168)	IF (1:200); WB(1:500)
α-tubulin (11H10)	Cell Signaling (2125S)	IF (1:200)
TOP2	Abcam (ab109524)	IF (1:200)
CREST	Fitzgerald Industries International (70R-21494)	IF (1:100)
BTG4	Abcam (ab206914)	WB (1:500)
CNOT7	Santa Cruz (sc-101009)	WB (1:200)
CyclinB1	Cell Signaling (4138)	WB (1:1000)
WEE2	Proteintech (55119-1-AP)	WB (1:500)
CPEB1	Proteintech (13274-1-AP)	WB (1:500)
pERK1/2	Cell Signaling (9101)	WB (1:100)
FLAG	Sigma (F3165)	IF (1:200); WB (1:1000); IP (1:200)
HA	Cell Signaling (3724)	WB (1:1000)
TPX2	Novus Biologicals (NB500-183)	IF (1:200); WB (1:1500)
Pericentrin	BD transduction laboratories (611814)	IF (1:300)
MSY2	Abcam (ab154829)	IF (1:500); WB (1:1000)
ERK1/2	Santa Cruz (sc-94)	WB (1:1000)
Phosphorylated RNA Pol II (Ser2P)	Abcam (ab5095)	IF (1:40000)

Table S3. Quality control of RNA-seq results (WT and ZAR1/2-deleted oocytes).

Sample	Total Reads	Mapping Efficiency	Uniquely Mapping Efficiency	ERCC percentage
WT-growing-Rep1	15,248,073	92.75%	82.26%	0.166%
WT-growing-Rep2	18,216,406	93.10%	82.91%	0.139%
Zar1/2$^{-/-}$-growing-Rep1	15,023,565	93.06%	84.42%	0.195%
Zar1/2$^{-/-}$-growing-Rep2	18,210,629	92.68%	84.95%	0.188%
WT-GV-Rep1	15,550,782	91.48%	82.23%	0.196%
WT-GV-Rep2	16,238,175	90.74%	82.65%	0.196%
Zar1/2$^{-/-}$-GV-Rep1	15,711,195	90.09%	82.81%	0.297%
Zar1/2$^{-/-}$-GV-Rep2	14,500,417	92.08%	84.27%	0.238%
WT-MII-Rep1	13,803,666	90.65%	83.06%	0.736%
WT-MII-Rep2	17,435,478	90.34%	83.76%	0.622%
Zar1/2$^{-/-}$-MII-Rep1	14,479,436	90.78%	84.35%	0.579%
Zar1/2$^{-/-}$-MII-Rep2	17,458,221	91.60%	85.85%	0.552%

Table S4. FPKMs of RNA-seq results (in a separate xlsx file).

Table S5. Spearman correlation coefficients among WT and *Zar1/2*$^{-/-}$ oocytes.

Genotype		Growing				GV				MII			
		**WT-1**	**WT-2**	**KO-1**	**KO-2**	**WT-1**	**WT-2**	**KO-1**	**KO-2**	**WT-1**	**WT-2**	**KO-1**	**KO-2**
**Growing**	**WT-1**	1.00	0.96	0.95	0.95	0.96	0.96	0.95	0.95	0.91	0.91	0.92	0.92
	**WT-2**	0.96	1.00	0.95	0.95	0.95	0.96	0.95	0.95	0.90	0.91	0.92	0.92
	***Zar1/2* KO-1**	0.95	0.95	1.00	0.97	0.94	0.94	0.95	0.95	0.89	0.89	0.92	0.92
	***Zar1/2* KO-2**	0.95	0.95	0.97	1.00	0.93	0.94	0.95	0.95	0.88	0.89	0.92	0.92
**GV**	**WT-1**	0.96	0.95	0.94	0.93	1.00	0.96	0.95	0.95	0.91	0.91	0.92	0.92
	**WT-2**	0.96	0.96	0.94	0.94	0.96	1.00	0.95	0.95	0.91	0.92	0.93	0.93

	***Zar1/2* KO-1**	0.95	0.95	0.95	0.95	0.95	0.95	1.00	0.96	0.90	0.90	0.93	0.93
	***Zar1/2* KO-2**	0.95	0.95	0.95	0.95	0.95	0.95	0.96	1.00	0.89	0.90	0.93	0.93
**Ovulated (MII)**	**WT-1**	0.91	0.90	0.89	0.88	0.91	0.91	0.90	0.89	1.00	0.95	0.91	0.92
	**WT-2**	0.91	0.91	0.89	0.89	0.91	0.92	0.90	0.90	0.95	1.00	0.92	0.92
	***Zar1/2* KO-1**	0.92	0.92	0.92	0.92	0.92	0.93	0.93	0.93	0.91	0.92	1.00	0.96
	***Zar1/2* KO-2**	0.92	0.92	0.92	0.92	0.92	0.93	0.93	0.93	0.92	0.92	0.96	1.00

Table S6. FPKMs of transcripts decreased or increased for more than 3 folds in *Zar1/2*$^{-/-}$ oocyte samples (in a separate xlsx file).
